# Examining the key features of specialist health service provision for women with Female Genital Mutilation/Cutting (FGM/C) in the Global North: a scoping review

**DOI:** 10.3389/fgwh.2024.1329819

**Published:** 2024-05-22

**Authors:** Juliet Albert, Mary Wells, Helen Spiby, Catrin Evans

**Affiliations:** ^1^ University of Nottingham and Division of Womens, Children and Clinical Support, Imperial College Healthcare NHS Trust (ICHNT), London, United Kingdom; ^2^ Nursing Directorate, Department of Surgery and Cancer, Imperial College Healthcare NHS Trust (ICHNT), Imperial College London, London, United Kingdom; ^3^ School of Health Sciences, Faculty of Medicine and Health Sciences, University of Nottingham, Nottingham, United Kingdom; ^4^ The Nottingham Centre for Evidence Based Healthcare, Faculty of Medicine and Health Sciences, University of Nottingham, Nottingham, United Kingdom

**Keywords:** Female Genital Mutilation/Cutting, Global North, reconstruction, deinfibulation, scoping review, obstetric, gynaecology, specialist service

## Abstract

**Background:**

Health care for women with Female Genital Mutilation/Cutting (FGM/C) in the Global North is often described as sub-optimal and focused on maternity care. Specialist FGM/C services have emerged with little empirical evidence informing service provision. The objective of this scoping review is to identify the key features of FGM/C specialist care.

**Methods:**

The review was conducted in accordance with JBI methodology. Participants: organisations that provide specialist FGM/C care. Concept: components of specialist services. Context: high-income OECD countries. Eligibility criteria included primary research studies of any design from 2012 to 2022, providing a comprehensive description of specialist services. Seven bibliographic databases were searched (MEDLINE, EMBASE, CINAHL, Web of Science, SCOPUS, Cochrane Library and MIC). The components of “specialist” (as opposed to “generalist”) services were defined and then applied to an analysis of FGM/C specialist care. FGM/C specialist provision was categorised into primary (essential) and secondary features. Data were extracted and analysed descriptively through charting in tables and narrative summary.

**Results:**

Twenty-five papers described 20 unique specialist services across eleven high income countries. Primary features used to identify FGM/C specialist care were:—(i) Named as a Specialist service/clinic: 11/20 (55%); (ii) Identified expert lead: 13/20, (65%), either Midwives, Gynaecologists, Urologist, or Plastic Surgeons; (iii) Offering Specialist Interventions: surgical (i.e., reconstruction and/or deinfibulation) and/or psychological (i.e., trauma and/or sexual counselling); and (iv) Providing multidisciplinary care: 14/20 (70%). Eleven services (in Spain, Sweden, Switzerland, Germany, Italy, Netherlands, France, Belgium, and USA) provided reconstruction surgery, often integrated with psychosexual support. No services in UK, Norway, and Australia offered this. Six services (30%) provided trauma therapy only; 25% sexual and trauma therapy; 15% sexual therapy only; 30% did not provide counselling. Secondary features of specialist care were subdivided into (a) context of care and (b) the content of care. The context related to concepts such as provision of interpreters, cost of care, community engagement and whether theoretical underpinnings were described. Content referred to the model of care, whether safeguarding assessments were undertaken, and health education/information is provided.

**Conclusion:**

Overall, the features and composition of FGM/C specialist services varied considerably between, and sometimes within, countries. Global guidelines advocate that specialist care should include access to deinfibulation, mental health support, sexual counselling, and education and information. The review found that these were rarely all available. In some high-income countries women cannot access reconstruction surgery and notably, few services for non-pregnant women mentioned safeguarding. Furthermore, services for pregnant women rarely integrated trauma therapy or psychosexual support. The review highlights a need for counselling (both trauma and psychosexual) and culturally-appropriate sensitive safeguarding assessments to be embedded into care provision for non-pregnant as well as pregnant women. Further research is needed to extract the features of specialist services into a comprehensive framework which can be used to examine, compare, and evaluate FGM/C clinical specialist care to determine which clinical features deliver the best outcomes. Currently a geographical lottery appears to exist, not only within the UK, but also across the Global North.

## 1 Introduction

Female Genital Mutilation/Cutting (FGM/C) is when the female genitals are deliberately cut or injured without medical reason, carrying lifelong negative health consequences (1). Globally, it is estimated that more than 200 million women (2), 5% of the female population, have suffered FGM/C. This is recognised as a human rights violation and form of gender-based violence. FGM/C is a global public health concern and presents an increasing challenge to countries in the Global North with large diaspora.

FGM/C is classified into 4 types by the WHO (see Table 1), and long-term sequelae include urological, gynaecological, and obstetric complications, sexual dysfunction and psycho-social issues (1). There are limited treatment options to relieve some of the physical symptoms. For common physical problems such as vulvodynia, clitoral pain and genital scarring, non-surgical treatments might include the use of water-soluble lubricants during sexual intercourse, local anaesthetics (such as lidocaine gel) or hormonal creams. Women also frequently report body image concerns and are at higher risk of HIV, hepatitis B or C from the use of non-sterile cutting equipment. In addition, pregnant women are at increased risk of prolonged labour, postpartum haemorrhage, perineal trauma, caesarean section, stillbirth and early neonatal death (3, 4). It is well reported that FGM/C may also have psychological consequences such as Post Traumatic Stress Disorder, flashbacks, nightmares, depression, anxiety and touch and needle phobia (4). Psychoeducation, Psychotherapy and/or pharmacological intervention may be offered. There may also be sexual health consequences such as dyspareunia, reduced sexual satisfaction and reduced sexual desire which can be treated with psycho-sexual counselling and potentially surgical interventions (4, 5).

**Table 1:** WHO classification of FGM/C types.

FGM/C type	Description
Type 1	Partial or total removal of the clitoral glans and/or the prepuce. Sometimes known as clitoridectomy.
Type 2	Partial or total removal of the clitoral glans and the labia minora, with or without excision of the labia majora. Sometimes known as excision.
Type 3	Narrowing of the vaginal orifice with creation of a covering seal by cutting and appositioning the labia minora and/or the labia majora, with or without excision of the clitoral glans. Sometimes known as infibulation or pharaonic circumcision.
Type 4	All other harmful procedures to the female genitalia for non-medical purposes, including Gishiri cuts, pricking, piercing, incising, scraping and cauterisation and labial elongation.

Adapted from WHO (1).

Two main surgical procedures may be offered to women who have suffered FGM/C depending upon the type. The first is deinfibulation. This opens the sealed vulva and exposes the vaginal opening and urinary meatus for women who have Type 3. This can be performed on non-pregnant women at any time, or pregnant women during pregnancy, intrapartum or perioperatively after caesarean section (3), by suitably trained Midwives, Nurses or Doctors. This does not replace missing tissue but does allow for sexual intercourse, childbirth, taking of cervical smears and relieves problems such as dysuria, apareunia or dyspareunia, dysmenorrhea surgical management of miscarriage etc. The second approach is reconstructive surgery. This aims to restore original genital appearance by revealing any remaining clitoral tissue and/or rebuilding the clitoral glans and/or clitoral hood and/or labia. Reconstructive surgery can be performed for non-pregnant women with Type 1,2, or 3 FGM/C by either plastic surgeons, urologists, uro-gynaecologists, or gynaecologists. At present, reconstruction surgery is only available in some countries in Europe, Africa, and parts of USA. (In this paper we shall refer to reconstruction surgery as one treatment approach rather than addressing the variations in technique that exist). Other surgical interventions may also be required to address voiding dysfunction, scarring and cysts secondary to the adverse effects of FGM/C.

In the UK (where the authors are based), there are an estimated 137,000 FGM/C survivors (6), predominantly from Black, Asian and Minority ethnic communities. Treatment for FGM/C-related conditions is estimated to cost the National Health Service (NHS) approximately £100 million annually (7). In 2013 an independent group of FGM/C experts, known as the FGM/C National Clinical Group, published recommendations for holistic FGM/C specialist services (8). They identified that psychological support was rarely provided and, when available, it was primarily short term, inaccessible to non-English speakers and trauma-focused (9). In addition to several existing FGM clinics (mostly within maternity services), in 2018 the National Health Service (NHS) commissioned five new clinics for non-pregnant women, providing a tripartite model of care based upon the Acton model (10–12). This co-located a specialist clinician, health advocate and counsellor, within a community setting, in areas of high prevalence (10, 12, 13). However, despite this initiative, a recent study found FGM/C service provision in the UK to be suboptimal (14). This scoping review was undertaken to inform a national (UK) study investigating the nature and experiences of FGM/C specialist care provision (as distinct from care provided in “generalist” settings). Examples of “generalist” care for FGM/C survivors might be a General Practitioner appointment (where FGM is discussed), standard care during pregnancy or childbirth, or a general gynaecological consultation. Review goals were identified in consultation with two Patient and Public Involvement (PPI) groups, one consisting of FGM/C survivors and one of Health Advocates (women from FGM/C-practising communities working in healthcare).

In the Global North, FGM/C specialist services have been established in many countries to treat the consequences of FGM/C, however, many of these services have been set up on an *ad hoc* basis, as an adjunct to maternity services. A review of the current research in this topic reveals a number of studies which have explored the healthcare experiences of women with FGM/C within “general” (i.e., non-specialist) healthcare settings (15–24). Few studies, however, have examined specialist care.

Guidelines introduced by the World Health Organisation (WHO) in 2016 recommend that all FGM/C survivors have access to deinfibulation, mental health support, sexual counselling, and education and information (25). In spite of this, in many countries services frequently remain orientated towards physical care in preparation for childbirth, and psychological support is rarely integrated into care pathways (26).Overall, the evidence suggests that there is significant variation in the configuration of FGM/C specialist services internationally (26). In addition, there is a lack of evidence regarding optimal models of specialist care (27). Existing services lack a theoretical basis and have often been developed pragmatically and organically in response to perceived needs (28). In addition, there is little evidence on the views of women and their partners in terms of how services can best be designed to meet women's complex needs (29, 30). In order to develop a better understanding of the optimal components and design of specialist care delivery, there is a need to characterise and explore specialist FGM/C services in more detail. To do this, it is important to make a distinction between generalist and specialist care for FGM/C survivors.

### 1.1 Rationale

In previous evidence reviews authors have distinguished FGM/C interventions for pregnant and non-pregnant women. Chappel (31) identified deinfibulation, cyst excision, clitoral and vulvar reconstruction, urological reconstruction, peripartum procedures, labial adhesion release, and reinfibulation as surgical interventions for FGM/C-related morbidity for non-pregnant women. In contrast, Balogun (32) examined maternal care interventions and identified:- deinfibulation; assisted delivery; infection and cyst treatment; psychological counselling; and health focused anti-FGM/C education.

A 2018 study by Johansen et al. presented an overview of FGM/C-related healthcare across 30 countries, including 11 countries of origin and 19 countries of migration (26). They identified four main treatment options: deinfibulation, psychological counselling, sexual counselling, and reconstruction. They found that, in some countries, deinfibulation was only available in the context of pregnancy and childbirth, or in private clinics. Reconstruction surgery was available in 10/19 migration countries. Eight migration countries legislated mandatory health education during FGM/C consultations; and several countries provided psychological and/or sexual counselling services, but there was significant regional variation (26).

A mixed methods study by Baillot et al. in 2018 (27) examined the provision of FGM/C services in Europe. The authors identified themes of access, reconstruction, and sustainability as key dimensions of FGM/C service provision. They recommended that services be co-designed using a culturally competent lens and highlighted the importance of community engagement and safeguarding (27). A scoping review published by Dawson et al. in 2022 (33) examined guidance and tools available to healthcare workers across six English speaking high income countries (UK, Ireland, Canada, Australia, US, New Zealand). The UK had produced the majority of publications, mostly focused upon multi-professional safeguarding. The authors also recommended that services be co-designed in order to be truly patient-centred. Evans et al.'s systematic review in 2019 (28) examined FGM/C service provision across high income countries and concludes that there remains a lack of standards, with commissioning arrangements varying considerably.

### 1.2 Aims and objective

The aim of this scoping review was to identify and describe the key features of FGM/C specialist service provision in high-income countries of the Global North. This included examining features such as service configuration, content of care and those characteristics which act as facilitators of access to services. By examining the key features of FGM/C-related *specialist care*, the review hopes to propose a framework which can be developed further to enable a more accurate analysis and comparison of what constitutes effective specialist care provision. To date, no studies or reviews have proposed or examined the features of a specialist service. This review seeks to fill this gap by elucidating the key features that FGM/C specialist services have in common (which thereby allows them to adopt the name “specialist”, and which distinguishes them from care provided in “general” settings). In doing so, it aims to identify the key components essential for high quality FGM/C specialist care.

## 2 Methods

A scoping review was the chosen method of evidence synthesis because it “*describes and maps a body of literature”* (34) and is suitable for undertaking broad searches in order to characterise a complex concept (35). The review was conducted in accordance with JBI methodological guidance (34) and the protocol was registered with the Open Science Framework (osf.io/gfzdm). The review is reported using the Preferred Reporting Items for Systematic Review and Meta-Analysis Extension for Scoping Reviews (PRISMA-ScR) (36) (see checklist in Supplementary File S1). A preliminary search of MEDLINE and the Cochrane Database of Systematic Reviews was conducted and no identical scoping reviews, either existing or in progress, were identified.

### 2.1 Operational definitions

In order to examine the features of FGM/C specialist services, it was first necessary to construct an operational definition for the term “specialist” service and how this can be conceptualised as distinct from “generalist”. The authors undertook a review of relevant health services literature (37–45). It was apparent that there was no single agreed or established definition of specialist care. In general however, the literature suggests that specialist services can be described as:- (i) Providing specialist care (i.e., providing care to patients with complex needs or focused on one specific area of need); (ii) led by healthcare professional(s) with advanced knowledge or expertise in a specific area of medicine; (iii) providing a variety of advanced treatments; and (iv) delivered by a multidisciplinary team. These concepts were referred to by the authors as the “primary features” of specialist care. These concepts also helped inform the key terms of the search strategy. All other features of specialist care provision, identified through literature searching and from reviewing the papers identified here, were then designated as “secondary features”. Secondary features were split into: (a) Contextual factors relating to the environment or setting of the healthcare service (46), and, (b) the content of care referring to a summary of the individual care model, including outcome measures and information/education provision.

The primary and secondary features were used to inform the data extraction and charting processes for the review (see below).

### 2.2 Eligibility criteria

Inclusion and exclusion criteria were defined utilizing the JBI Population-Concept-Context “PCC” framework (34), as shown in Table 2. A date limit of 2012–2022 was chosen to reflect a contemporary/current picture of service provision. There were no language restrictions.

**Table 2:** JBI “PCC” framework.

Participants/population
Participants were organisations that provide specialist FGM/C care. This included both NHS/free/public and private services, specifically for adult women, (excluding paediatric care for non-pregnant girls under 18 years).
Concept
Concept referred to specialist FGM/C service provision including components such as service configuration, theoretical underpinnings, treatments/therapies (trauma or psychosexual therapy), surgery/procedures (e.g., deinfibulation, labial and/or clitoral reconstruction), follow-up/outcome measures, care and referral pathways and accessibility. Evidence sources that included any of these terms, or which addressed similar concepts, were included.
Context
Context included all OECD countries in the Global North. These are countries that are “destination” countries for migrant women with FGM/C. The rationale for this approach is that the baseline health and care needs of FGM/C survivors from other high-income countries would be similar to that of survivors in the UK as they have access to comparable systems of care. Studies relating to middle and low-income countries were excluded.

### 2.3 Types of evidence

Inclusion and exclusion criteria were developed to ensure all publications with a clear and comprehensive description of service provision were incorporated in the review. Included studies were those presenting primary data: peer reviewed primary research, service evaluations, audits and unpublished evidence. Sources included quantitative, qualitative, and mixed methods designs, irrespective of methodological approach. Excluded studies were systematic or literature reviews relying upon secondary data sources, studies that included healthcare providers' attitudes and experiences only, with limited or no description of a specialist service or care pathway. Commentaries, study protocols and conference abstracts were excluded at the time of full text screening because they did not present sufficient primary data referring to the population of interest.

### 2.4 Search strategy

#### 2.4.1 Information sources

A search strategy was carried out using the JBI three step process of search strategy development (47). In step (1), the first reviewer (PhD student/JA), with professional librarian support, searched Ovid MEDLINE(R) ALL <1946 to September 01, 2022 > MEDLINE to identify initial key search terms, which included synonyms for FGM/C, such as female circumcision, female genital cutting (FGC), excision, infibulation, ritual cutting, ritual circumcision, pharaonic circumcision, sunna circumcision and clitoridectomy. Search terms and concepts were identified using JA's experiential knowledge and further clarified with reference to publications by Dawson et al. (33), Baillot et al. (27), Evans et al. (48) and World Health Organisation (WHO) (49). Additional keywords and MeSH terms, such as person, woman and patient-centred care, were subsequently added. Injury of genitalia, labia and clitoris were removed, and the phrase excision was combined with wom?n/female*/girl* and genital*/labia*/clitor* to reduce the number of citations related to male circumcision (see Search Terms in Supplementary File S2). Papers by Albert and Wells (10) and Caillet et al. (50). were identified as seminal papers describing specialist FGM/C care that should be identified by the search strategy. Other index terms/keywords contained in the titles and abstracts of relevant articles were added.

In step (2), database-specific searches were adapted for each included database. The following electronic bibliographic databases of peer reviewed literature were reviewed: MEDLINE (Ovid SP), EMBASE (OvidSP), Web of Science, SCOPUS, Cochrane Library, CINAHL, and the Maternity and Infant Care (MIC) to ensure a broad spectrum of publications were included from both medical and allied health professional journals (see Search Strategy example in Supplementary File S3).

In step (3), the reference lists of sources of evidence were hand searched and cross checked for further articles of relevance. Authors were contacted to retrieve further information, where for example only a conference abstract had been published, or to retrieve a topic guide or access a full text. Supplementary search approaches also included contacting other researchers in the field via specialist networks and Google Scholar searching.

### 2.5 Evidence screening and selection of sources of evidence

Initial screening was conducted in English. Google Translator (Google, Mountain View, California, USA) was used if required, as validated by Balk et al. (51). Non-English articles which met the inclusion criteria were translated by contacting the author to request an English version or asking colleagues who are native speakers. Final search results were exported into EndNote 20 (Clarivate Analytics, PA, USA) and duplicates were removed. These were imported into Covidence systematic review software (Veritas Health Innovation, Melbourne, Australia. Available at www.covidence.org.), to facilitate screening and data extraction.

JA undertook the initial title and abstract screening to find articles that potentially met the inclusion criteria, followed by full text screening in consultation with the wider team. Full texts were assessed against the inclusion criteria. Reasons for exclusion were recorded (see Supplementary File S4). Excluded papers were mainly evidence reviews, interviews with women or healthcare professionals relating to experiences of general healthcare (rather than that of specialist care), and commentaries or recommendations.

Any uncertainty was discussed with the whole team (CE, MW and HS) at each stage of the selection process and resolved through discussion.

After final screening, each article was read and re-read repeatedly to identify key themes (52). Extracted data were presented and summarized into tables, with narrative summaries used to elaborate issues in more detail.

### 2.6 Data extraction, charting and analysis

A data extraction template (see Supplementary File S5) was developed iteratively by JA to determine which variables to extract. This was piloted and reviewed by the research team. The data extraction template was separated into four sections:—1. Study characteristics; 2. Primary features of specialist care; 3. Secondary features of specialist care and 4. Summary of key features. The template also drew upon elements from the Template for Intervention Description and Replication (TIDieR) framework (53) as well as key information identified from analysis of core sources (27–29, 31, 33, 48), experiential knowledge of JA, (an FGM/C specialist service lead), the research team and PPI.

Charting of study characteristics included basic information such as 1st author and year of publication. Data items for primary features of specialist care included whether the service:—is (i) referred to as specialist; (ii) has a named expert lead; (iii) offers at least one specialist intervention; and (iv) has multidisciplinary staff. Secondary features were divided into (a) context of care, and (b) content of care. Contextual factors relating to the environment or setting of the healthcare service were defined as:—eligibility criteria; referral pathway; cost of service; theoretical underpinnings; and whether the service was advertised. We had also intended to extract information relating to commissioning arrangements, but this heading was removed due to a dearth of information across studies. Data related to content of care included a summary of the individual care model, including outcome measures and information/education provision.

#### 2.6.1 Appraisal

Critical appraisal of individual sources of evidence was not undertaken, as the aim of the review was to describe and map services rather than appraise the quality of the evidence (35).

## 3 Results

### 3.1 Presentation of findings

#### 3.1.1 Results of literature search, screening and selection of sources of evidence

**Figure 1:**
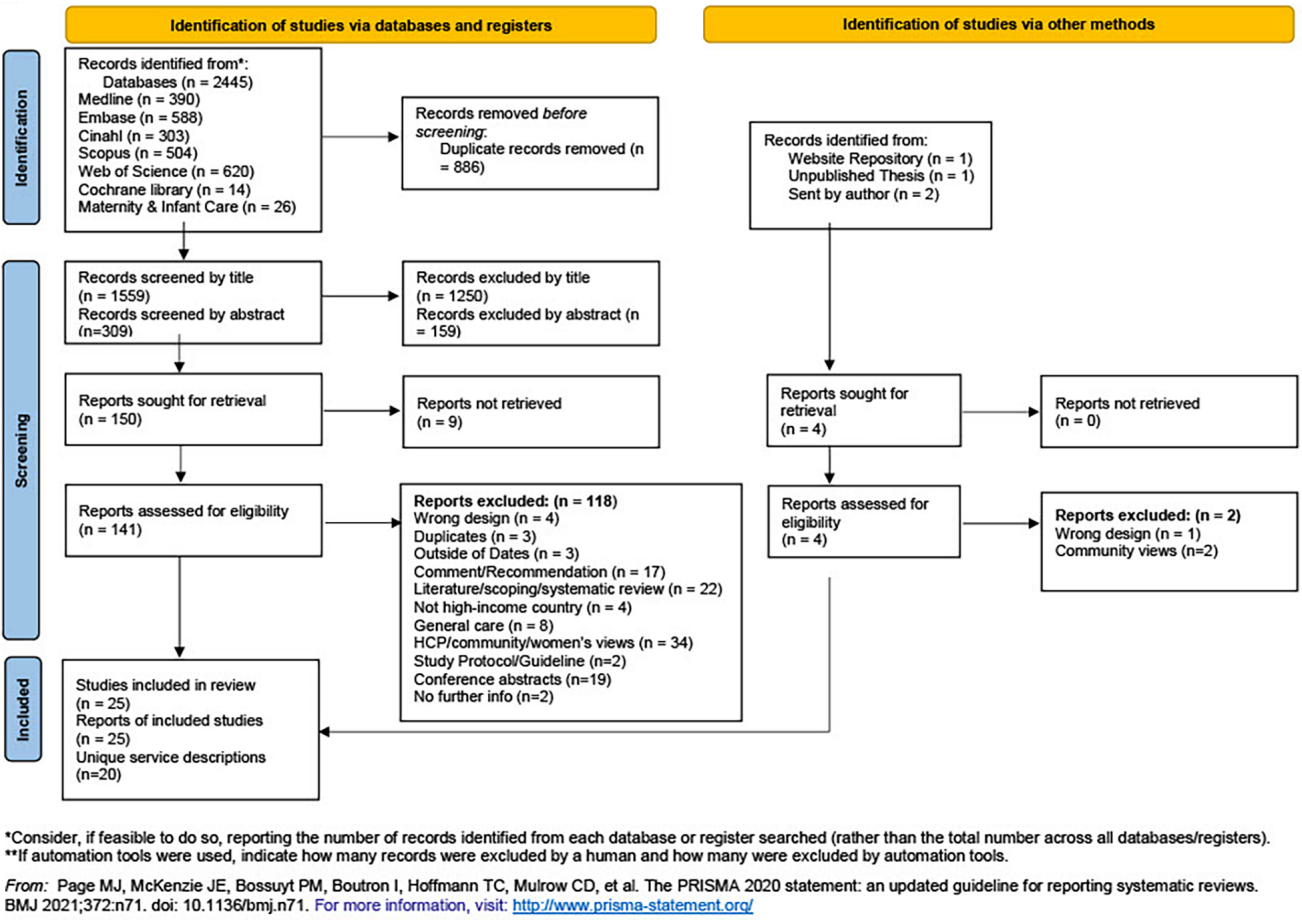
PRISMA flowchart.

A total of 2,445 articles were initially identified (see Figure 1). After removal of 886 duplicates, 1,559 articles were included. After screening by title and abstract, 150 studies remained for full-text eligibility. Four further studies were identified through supplementary searches. Therefore 154 papers were included for full text review.

After full text screening 129 studies were excluded (see Supplementary File S4 for a full list with reasons). Twenty five papers were included for final data extraction displayed in the (PRISMA-ScR) flow diagram (36), representing 20 unique services (See Figure 1).

#### 3.1.2 Synthesis and presentation of results

The main review findings are presented in four tables (Tables 3–6). The table presenting the content of care is a supplementary File S6 (no. 6) due to size restrictions. The first table (Table 3) displays general study characteristics from each paper to illustrate the sources of evidence. The second table (Table 4) presents the primary features of specialist service provision. The third table (Table 5) presents data on the secondary features (a) context of care. The fourth table (Table 6) presents a summary of all key features of specialist services, providing an easy reference table with which to compare respective services.

**Table 3:** Study characteristics.

#	Lead author; year of publication; reference	Name of service	Country where study conducted	Focus of paper	Study Design/Methodology/Methods	Total number of study participants	Start & End year of study	Number assigned in subsequent tables
1	Abdulcadir et al., 2016 (54)	Geneva University Hospital (HUG)—maternity	Geneva, Switzerland	Evaluation of Obstetric care	Retrospective review of medical records *Maternity service described	84 pregnant women out of total 129	2010–2012	HUG—maternity, #1
2	Abdulcadir et al., 2012 (55)	Geneva University Hospital (HUG)—Gynaecology	Geneva, Switzerland	Management of Clitoral neuroma	Retrospective case report study *Gynaecology service described	1 non-pregnant woman	NR	HUG—gynaecology, #2 (merged)
3	Abdulcadir et al., 2015 (56)	Geneva University Hospital (HUG)—Gynaecology	Geneva, Switzerland	Describes mdt care + clitoral reconstruction	Retrospective review—case reports *Gynae service described	2 non-pregnant women	NR	HUG—gynaecology, #2 (merged)
4	Abdulcadir et al., 2017 (57)	Geneva University Hospital (HUG)—Gynaecology	Geneva, Switzerland	Management of clitoral neuroma (some had clitoral reconstruction)	Retrospective review—case series *Gynae service described	7 non-pregnant women	2010–2016	HUG—gynaecology, #2 (merged)
5	Akhavan et al., 2020 (58)	NR	Sweden	Range of Swedish healthcare	Survey of ICD codes and literature review	6,216 women. Mostly pregnant	2012–2018	Akhavan #3
6	Albert and Wells, 2020 (12)	The Sunflower Clinic	London UK	Care for non-pregnant women	Retrospective review of medical records *Gynae service described	88 non-pregnant women	2018–2019	Sunflower clinic, #4
7	Beltran et al., 2015 (59)	NR	Bicetre, Paris, France	Sexological care for women with FGM/C	Retrospective review—2 case studies	2 non-pregnant women	NR	Bicêtre, #5
8	Caillet et al., 2018 (50)	Medical Centre in Aid of Victims of Excision (CeMaViE),	Brussels, Belgium	MDT care for women who request clitoral reconstruction	Service description	107 women	2014–2018	CeMaViE #6 (Merged)
9	Christopher et al., 2022 (60)	Division of Plastic and Reconstructive Surgery	Pennsylvania, Philadelphia, USA	Evaluation of patients who underwent clitoral & labial reconstruction	Medical records review & post-op survey	19 adult patients	2016-2021	Pennsylvania clinic, #7
10	Di Rosa, 2023 (61)	INSIGHT	Scotland, UK	Evaluation of Maternity care	Semi-structured interviews with stakeholders	12 pregnant women	2022	INSIGHT, #8
11	Dugast et al., 2017 (62)	Nantes teaching hospital	France	Evaluation of women who had sexology and/or clitoral reconstruction	Cross-sectional descriptive study—retrospective analysis of questionnaires	82 women	2011–2015	Nantes Teaching Hospital, #9 (merged)
12	Foldes et al., 2012 (63)	Poissy-St Germain Hospital	France	Evaluation of clitoral reconstruction	Retrospective review of medical records	2938 women	1998–2009	Foldes clinic, #10
13	Jordal et al., 2021 (64)	Karolinska University Hospital,	Stockholm, Sweden	Evaluation of clitoral reconstruction	Qualitative semi-structured interviews	18 non-pregnant women	2016–2019	Karolinska University Hospital #11
14	Karim et al., 2022 (65)	Department of Gynaecology and Obstetric,	Amsterdam, Netherlands	Outcomes of clitoral reconstruction (some had labial reconstruction and deinfibulation)	Retrospective review of medical records and questionnaires	45 women	2010–2020	Amsterdam #12
15	Manero and Labanca, 2018 (66)	Ivan Manero Clinic,	Barcelona, Spain	Describes clitoral reconstruction using vaginal graft to create labia minora	Quantitative study—validated assessment tools	32 women	2013–2016	Manero clinic, #13
16	Mestre-Bach, 2018 (67)	Dexeus University Hospital, Private hospital	Barcelona, Spain	Changes in Sexual Distress, Depression and Sexual Function after Clitoral Reconstruction	Quantitative Longitudinal study—use of validated assessment tools	27 non-pregnant women	NR	Dexeus, #14 (Merged)
17	O’Neill et al., 2022 (68)	Medical Centre in Aid of Victims of Excision CeMaViE,	Brussels, Belgium	Describes two women who requst clitoral reconstruction but decide against it	Qualitative Participant observation, informal conversations, and in-depth interviews	2 non-pregnant women	2017–2019	CeMaViE,#6 (Merged)
18	Paliwal et al., 2014 (69)	Birmingham Heartlands Hospital	Birmingham, UK	Obstetric care of women with Type 3	retrospective case analysis using patient records * Maternity care pathway described	253 pregnant women	2008–2009	Birmingham Heartlands, #15
19	Paslakis et al., 2020 (70)	Dexeus University Hospital, Private hospital	Barcelona, Spain	Evaluation before & after reconstruction surgery	Pilot longitudinal study—15 women attended 1 year follow up	43 women	2015–2020	Dexeus, #14 (Merged)
20	Restaino et al., 2022 (71)	Institute for Maternal and Child Health—IRCCS Burlo Garofolo	Trieste, Italy	Woman has deinfibulation + clitoral reconstruction	Retrospective review of medical records—one case study	1 woman	NR	IRCCS, #16
21	Shukralla and McGurgan, 2020 (72)	Specialist FGM/C clinical care pathway	2 Perth hospitals, Australia	Maternity care audit	Audit of medical records	53 pregnant women	2014	Perth hospitals, #17
22	Varol et al., 2016 (73)	Sydney metropolitan hospital	Sydney Australian	Obstetric outcomes	Retrospective review of medical records	196 pregnant women	2006–2012	Sydney metropolitan, #18
23	Vital et al., 2016 (74)	Nantes teaching hospital. Outpatient clinic	Nantes, France	Evaluating sexual function before & after clitoral reconstruction	Observational, prospective, single-centre pilot study	12 women	2013-2014	Nantes Teaching Hospital, #9 (merged)
24	Zenner et al., 2013 (75)	University College London Hospitals	London, UK	Care for pregnant women	Retrospective review	39 pregnant women	2009	UCLH, #19
25	Ziyada and Johansen, 2021 (76)	NR	Norway	Access to specialist healthcare	Qualitative in-depth semi-structured interviews & focus group	43 non pregnant women	2016–2018	Norway, #20

**Table 4:** Primary features characterising specialist services.

Number	Name of service Publication references	Described as specialist service?	What interventions provided?	Is there a named service lead	Who provides care?
#1	HUG—maternity (54)	“Specialized clinic in a tertiary centre”	Prenatal consultation; FGM/C diagnosis & counselling re:- timing of defibulation post-partum perineal physiotherapy if woman requests reinfibulation; Family-planning	Gynaecology expert lead	Specialized FGM/C gynecologist- lead Red Cross professional interpreters Multidisciplinary working group Psychosomatic Gynaecology and Sexologist
#2 (merged)	HUG—gynaecology (55–57)	Specialized clinic for women with FGM/C	Clitoral reconstruction (Foldes technique) Surgical excision of cysts excision of painful mass Deinfibulation Histology of the peri clitoral fibrosis removed during surgery	Gynaecology expert lead	Specialized FGM/C gynecologist- lead; Sexologist; Reproductive and sexual health counselor. Paediatrics; Forensic science expert, violence against women experts, medical anthropology, law, sexual therapy, psychiatry, psychology
#3	Akhavan (58)	2 specialist medical centres	Vulva plastic surgery, perineum reconstruction. Deinfibulation. Clitoris reconstruction, and removal of cysts.	NR	Female gynaecologists, midwives, and “curators” Plastic surgeon Female professional interpreter Psychosexual counselling
#4	Sunflower clinic (10)	Specialist service for non-pregnant women	FGM/C diagnosis. Deinfibulation under local anaesthetic. Fast track referral for deinfibulation under general anaesthetic;	Midwife-led	Named link consultant; Health advocates—Somali and Arabic speakers; Link to local NGO; Trauma psychotherapist;
#5	Bicêtre (59)	“Care unit for women with FGM/C”	Gynaecological, psychological and sexological support.	NR	Midwife, gynaecologist, sexologist
#6 (merged)	CeMaViE (50, 68)	“Medical Centre in Aid of Victims of Excision (CeMaViE)” specialist clinic	Clitoral reconstruction surgery, (Foldes technique modified by Ouedraogo) Psychotherapy Sexual therapist Medical certificate	NR	Gynaecologist Expert Midwife, Psychotherapist & Sexologist
#7	Pennsylvania service (60)	NR	Labial and clitoral reconstruction Donor site surgery Cyst removal	Plastic Surgeon	Plastic Surgeon—Plans to become a multidisciplinary specialist service
#8	INSIGHT (61)	INSIGHT specialist care pathway for pregnant women	Deinfibulation Contraceptive support Paediatric support and examination.	Midwife-led	Midwife-led Gynaecologist Child Protection Advisor 5 Insight team members 8 Community Midwives
#9 (merged)	Nantes Teaching Hospital (77, 78)	multidisciplinary “tailored’’ care pathway in the Medico-Psycho-Social Obstetrics Gynaecology Unit	Clitoral reconstruction Sex therapy Psychotherapy	Gynaecologist	Gynaecology Surgeon. Sexologist. Forensic Medicine Social Worker. Interpreters Psychotherapist
#10	Foldes clinic (63)	A Urology clinic	Clitoral reconstruction Deinfibulation and removal of pseudocysts	Urologist	Urologist—Plans to become a multidisciplinary specialist service
#11	Karolinska University Hospital (64)	Plastic surgery Clinic receives referrals from a specialised clinic. “Specialist care”	Clitoral Reconstruction (modified Foldes technique). Women with T3 undergo deinfibulation and Clitoral Reconstruction in 2 steps. Removal of cysts	Plastic Surgeon	Plastic Surgeon Psychosexual therapist
#12	Amsterdam service (65)	NR	Clitoroplasty (Foldès technique); Labia reconstruction Deinfibulation. Cyst removal. Treatment for urethral meatus stenosis	NR	Gynaecologist, Plastic surgeon, Case manager, Sexologist Female nurse
#13	Manero service (79)	“a single centre with a dedicated surgical team with considerable experience in reconstruction’	Novel surgical technique for clitorolabial reconstruction using a vaginal graft.	Plastic Surgeon	Plastic surgery team
#14 (merged)	Dexeus service (70, 80)	specialist care pathway	clitoral reconstruction and psychoeducational intervention	Gynaecologist	FGM/C expert Gynaecologist & clinical psychologist from Dept of Psychiatry, Psychology and Psychosomatic
#15	Birmingham Heartlands (69)	Specialist FGM/C service within maternity department	Deinfibulation under local anaesthetic FGM/C diagnosis Counselling re timing of defibulation	Midwife-led	Midwife-lead Access to named/link consultant
#16	IRCCS (71)	“Regional project for FGM/C and women immigrants”	Clitoral Reconstruction under general anaesthetic ‘Therapeutic Deinfibulation’	Plastic surgeon	Plastic surgeon Gynaecologist-Obstetrician Psychologist Cultural mediator
#17	Perth hospitals (72)	Specialist FGM/C clinical care pathway	FGM/C Diagnosis Intrapartum deinfibulation (no mention of whether offered antenatal deinfibulation)	NR	Senior Doctor (registrar or consultant) for birth plan Social worker referral Access to interpreters
#18	Sydney metropolitan (73)	Specialist FGM/C clinic	Antenatal or intrapartum deinfibulation. Counselling translation services	Clinical Midwife consultant	Specialist team of Midwives and Obstetricians & Clinical Midwife Consultant
#19	UCLH (75)	Specialist FGM/C clinic—maternity service described	Deinfibulation as an outpatient procedure Psychologist	Gynaecologist	Specialist obstetrics-gynaecology consultant psychological advice. Professional interpreters
#20	Norway service (76)	Low-threshold specialized healthcare services for pregnant & non-pregnant women.	Deinfibulation and removal of cysts.	NR	Gynaecologist Midwife Interpreters Specialized NGOs Social workers.

**Table 5:** Secondary features—(a) the context of care.

	Eligibility	Referral pathway	Number of clients seen	Cost	Theoretical underpinning	Advertising
HUG maternity clinic, #1	Pregnant women	NR	129 women between April 2010 and April 2012	NR	“Culturally sensitive care”	NR
HUG gynaecology clinic, #2	Non-pregnant women	NR	Between 2010 and 2016 clinic has seen approx. 15 women per month	NR	Holistic Multidiscplinary care	NR
Adkhavan #3	Both pregnant & non-pregnant	NR	6,216	NR	NR	NR
Sunflower clinic, #4	Non-pregnant. No geographical limit >18 years old;	All referrals accepted. E.g. Self-referral General Practitioner Other hcps GO's Lawyers Social workers	808 new clients between 2008 and 2019 *Information from unpublished paper	Public health—Free	Truama- informed Culturally sensitive Holistic Multidisciplinary Woman-centred	Advert for Somali satellite television
Bicêtre, France #5	NR whether pregnant or non-pregnant	NR	100 women per year	NR	Multidiscplinary Symbolic, aesthetic, and cultural repair	NR
CeMaViE, #6	NR whether pregnant or non-pregnant	Mainly referred by refugee centres, NGOs and medical doctors. *info taken from conference abstract (81)	667 women between 2014 and 2017.* info taken from conference abstract (81)	Costs reimbursed by Belgian social security	Multidisciplinary Holistic Trauma-informed Individualized care	NR
Pennsylvania, #7	NR whether pregnant or non-pregnant >18 years	NR	NR	NR	NR	NR
INSIGHT, Scotland, #8	Pregnant women only	Identified at booking	NR	Free	Cultural competence Patient-centred care	NR
Nantes Teaching Hospital, #8	NR whether pregnant or non-pregnant	NR	82 women from 2011 to 2015	free since 2004	NR	NR
Foldes clinic, #10	NR whether pregnant or non-pregnant >18 years with Type 2 or Type 3 FGM/C Patients with Type 3 without excision of the clitoris are excluded	Most patients present themselves, but some are referred	2938 women between 1998 & 2009 (6,000 women between 1998 and 2017)	free since 2004 publicly funded healthcare system	NR	Service not publicised, but in 2004 gained publicity (newspaper/ television) after decision by French health-care system to reimburse surgery
Karolinska University Hospital, #11	NR whether pregnant or non-pregnant Only women who convince their gynaecologists they have the ‘right’ motivations and expectations, demonstrating ‘maturity’, undergo CR	Women seek referral via primary healthcare, or their own gynaecologist or a specialized clinic. Sometimes difficulty gaining access because their gynaecologist refused to do referral.	Since 2014, more than 40 women have undergone surgery	NR	self-categorization and labelling theory ‘bioprecarity’	Women heard about the availability of CR through the radio, TV, newspapers, the Internet, a specialist FGC clinic, their GP or friends.
Amsterdam, #12	NR whether pregnant or non-pregnant Women ineligible for FGM/C corrective surgery, if: (i) FGM/C type inconsistent with extent, severity, or type of mutilation described or if patient expressed unrealistic expectations	Patients self-refer, but currently are mostly referred by a general practitioner or gynaecologist	45 women had genital reconstruction surgery between 2010 and 2020	Not specified but authors state *“it should be covered by health insurance"*	culturally sensitive approach	NR
Manero clinic, #13	NR whether pregnant or non-pregnant Exclusion criteria were patients who had undergone any other aesthetic surgery in their genitals.	NR	32 women between 2013 & 2016	NR	NR	NR
Dexeus, #14	NR whether pregnant or non-pregnant	Referred through their primary health care physicians or sought treatment themselves	43 women between 2015 & 2020	Private hospital funded by a charity	NR	NR
Birmingham Heartlands, UK #15	Pregnant women	Pregnant women asked at antenatal booking appointment	253 women between 2008 & 2009	Public health—free	NR	NR
IRCCS, #16	NR whether pregnant or non-pregnant	15 women that came in our Hospital spontaneously.	NR	NR	Culturally sensitive care	NR
Perth hospitals, #17	Pregnant women	Asked at booking	53 women in 2014	Public health—free	Culturally sensitive care	NR
Sydney metropolitan, #18	Pregnant women	Pregnant women asked at a booking appointment	196 women between 2006 & 2012	Public health—free	Cultural competence Holistic	NR
UCLH, #19	Pregnant women	Pregnant women asked at booking	39 Black African women with FGM/C over a 6-month period in 2009	Public health—free	NR	NR
Norway, #20	NR whether pregnant or non-pregnant	Access to specialist healthcare requires a referral from primary healthcare level. Some hospitals allowed women to self-refer.	358 women & girls during a 10–12-year period	Patients pay subsidized fee when visiting GP & outpatient specialists. Payments between 18 and 40 USD	Candidacy theory	Several websites provide info on services in various languages. Primary source of info was seminars arranged by non-profit immigrant organizations.

NR, not reported; Y, Yes; X, No).

**Table 6:** Summary of primary and secondary features.

		Offers *R* = reconstruction *D* = deinfibulation *S* = sex therapy *T* = trauma therapy		MDT	Calls itself a specialist service	A defined Service lead	*P*–pregnant NP = non-pregnant *B* = both	Safe-guarding	Education	Interpreter	Community engagement	Theoretical underpinning
1. 1	HUG—maternity, Switzerland	D	ST	✓	✓	✓	P	✓	✓	✓	✓	✓
2. 2	HUG—gynaecology, Switzerland	RD	ST	✓	✓	✓	NP	NR	✓	NR	NR	✓
3.	Akhavan, Sweden	RD	S	✓	✓	NR	B	NR	X	✓	NR	NR
4.	Sunflower clinic, UK	D	T	✓	✓	✓	NP	✓	✓	✓	✓	✓
5.	Bicêtre, France	X	ST	✓	✓	NR	NR	NR	✓	NR	NR	✓
6.	CeMaViE. Belgium	R	ST	✓	✓	NR	NR	✓	✓	✓	✓	✓
7.	Pennsylvania, USA	R	X	NR	NR	✓	NR	NR	NR	NR	NR	NR
8.	INSIGHT, UK	D	T	✓	pathway	✓	P	✓	✓	✓	NR	✓
9.	Nantes Hospital, France	R	ST	✓	pathway	NR	NR	NR	✓	NR	NR	NR
10.	Foldes clinic, France,	RD	X	NR	NR	✓	NR	NR	NR	NR	NR	NR
11.	Karolinska University Hospital, Sweden	RD	S	✓	✓	✓	NR	NR	✓	NR	NR	✓
12.	Netherlands	RD	S	✓	NR	NR	NR	NR	NR	NR	NR	✓
13.	Ivan Manero clinic, Spain	R	X	NR	NR	NR	NR	NR	NR	NR	NR	NR
14.	Dexeus, Spain	R	T	✓	pathway		NR	NR	✓	NR	NR	NR
15.	Birmingham Heartlands, UK	D	X	NR	✓	✓	P	✓	x	✓	NR	NR
16.	IRCCS, Italy	RD	T	✓	NR	NR	NR	NR	✓	NR	✓	✓
17.	Perth hospitals, Australia	D	X	✓	pathway	NR	P	✓	NR	✓	NR	✓
18.	Sydney metropolitan, Australia	D	T	✓	✓	✓	P	✓	✓	✓	NR	✓
19.	UCLH clinic, UK	D	T	✓	✓	NR	P	✓	NR	✓	NR	NR
20.	Norway	D	X	✓	✓	✓	✓	NR	NR	✓	NR	✓

NR, not reported; ✓, Yes; X, No).

When analysing the included studies, it became apparent, that in some cases more than one publication (sometimes by different authors) referred to the same specialist service. For example, four separate articles described the HUG clinic in Geneva. The data charting and analysis needed to take this into account. For this reason, Table 3 (study characteristics), presents data from each individual paper (*n* = 25). However, the remaining tables present data in relation to each unique service (*n* = 20).

### 3.2 Characteristics of included studies

Table 3 summarises study characteristics of the 25 included papers. Participant details, such as ethnic origin, FGM/C Type, and age, were not deemed relevant to this review and are not reported.

Included studies comprised twenty four journal articles, and one unpublished thesis (61). Publication dates ranged from 2012 to 2022. Papers were from the following countries: Switzerland (*n* = 4), Sweden (*n* = 2); UK (*n* = 4); France (*n* = 4); Belgium (*n* = 2); USA (*n* = 1); Netherlands (*n* = 1); Spain (*n* = 3); Italy (*n* = 1); Australia (*n* = 2) and Norway (*n* = 1). Sample sizes ranged from 1 to 6,216. The largest sample reported Swedish hospital ICD codes (58). Six papers (24%) were case study reports or case series with sample sizes of less than 10 women (of which two papers had one participant and three had two participants). There was one service description, one audit, and two service overviews. The others were all primary research. Fourteen papers (14/25, 56%) were retrospective reviews of medical records. Most of the papers were observational studies containing limited service-level detail. No chapters from textbooks were found to provide primary data but one textbook commented on the social and political context of service commissioning and the influence of professional positioning in the UK. This will be explored further in the discussion (82).

Of these 25 papers, 20 unique service descriptions were identified. Of these, one paper described care provided by two specialist FGM/C medical centres in Sweden (58), one described the national provision of low-threshold (being services that did not require GP referral) specialized FGM/C healthcare services in Norway (76) and one was an audit of FGM/C care offered by two maternity hospitals in Perth, Australia (72). The other 17 papers related to individual unique services within countries. Within this paper, services are referred to by the name of the clinic if clearly given or by the location. If information regarding the location or name of service is lacking, then the first author's name is used.

Where more than one paper described one service, these were merged as per the following process. Four papers pertained to the HUG clinic in Geneva Switzerland (54–57). The first study describes maternity care in the HUG clinic and is referenced as #1 “HUG maternity” in subsequent tables. The three Abdulcadir papers (numbered 2, 3, and 4) describe care for non-pregnant women in the HUG clinic. These are merged to become #2, “HUG gynaecology” (see Table 5 below). Two papers describing the Belgium CeMaVIE (50, 68) were similarly merged, two describing the Nantes Teaching Hospital in France (74, 77) and two describing the Dexeus service (Spain (70, 80) respectively.

### 3.3 Key features characterising specialist services

#### 3.3.1 Primary features

Table 4 displays the primary features characterising the 20 unique specialist services merged from Table 3. Primary features, as described earlier, were identified as:—(i) services named as specialist; (ii) Identified expert lead; (iii) offered specialist treatments; and (iv) involved multidisciplinary team members.

##### 3.3.1.1 Identification as a specialist service

With reference to Table 4, eleven service descriptions (11/20, 55%) used the phrase “specialist service” or “specialist clinic”. Four (4/20, 20%) referred to a unique care “pathway”, (three used the word “specialist” to describe the pathway and one was described as a multidisciplinary ‘‘tailored’' care pathway). Notably, two out of the four “pathways” were providing maternity care. Those services not specified as “specialist” were identified by this review for the following reasons. The Bicetre, France service calls itself a “care unit for women with FGM/C” (59). The Karolinska university hospital, Sweden “*receives referrals from a specialist clinic”* (64). The Manero, Spanish service is a “*single centre with a dedicated surgical team”* (66) and the paper describing the service in Italy refers to “*a regional project for FGM/C and women immigrants”* (71). The final three services were included because they contained other elements from the defined primary features (i.e., two papers described the provision of specialist surgery and stated an intention to become multidisciplinary services (60, 63) and one study from the Netherlands clearly described a multidisciplinary team approach to care with multiple treatment options (65).

##### 3.3.1.2 Identified expert lead

Clinical leads were either Midwives (*n* = 4), Gynaecologists (*n* = 4), Urologist (*n* = 1), Plastic Surgeons (*n* = 4) or not explicitly stated (*n* = 7). Both the HUG and Dexeus clinics refers to the gynaecologist as the lead “expert”. Three of the four UK services were Midwife-led as well as the Sydney hospital, Australia. All Midwives reported working with a link/named medical consultant.

##### 3.3.1.3 Provision of at least one specialist intervention

All 20 service descriptions offered at least one surgical or one psychological intervention as well as other non-surgical treatments/support. Various interventions were described, including deinfibulation, reconstruction surgery with different techniques, psychosexual therapy, trauma counselling, cyst excision, FGM/C diagnosis, perineal physiotherapy, family planning and treatment for urethral meatus stenosis. One paper referred to “psychoeducation” as an intervention. Eleven (55%) services offered reconstruction surgery (50, 56, 57, 60, 62–68, 70, 71, 74, 78, 80) located in Spain, Sweden, Switzerland, Germany, Italy, Netherlands, France, Belgium, and USA. One author presented two case studies from the Belgium clinic where the women requested reconstruction surgery but then decided against it after counselling (68); one paper (71) described a woman who underwent clitoral reconstruction with deinfibulation; and another paper described the care for seven women with clitoral neuroma, six whom had reconstruction as part of their excision surgery (57). Three services (60, 65, 79) offered labial as well as clitoral reconstruction. Jordal et al. (64) recommended a 2 step process of deinfibulation followed by reconstruction (this is likely to be because women might, after deinfibulation, no longer want reconstruction surgery). Jordal (Karolinska, Sweden) pointed out that women wanted labial reconstruction (as well as clitoral), but this was not available (64). Four studies (50, 59, 76, 83) commented that after education and psychosexual support, women might not choose reconstruction surgery. None of the six services located in UK, Norway, and Australia offered reconstruction (10, 61, 69–75, 72). All six maternity services and 8/20 (40%) of the services for non-pregnant women (including one service for both pregnant and non-pregnant women) offered deinfibulation as a treatment option. Reports of five services did not mention deinfibulation.

##### 3.3.1.4 Provision of a multidisciplinary team

Sixteen of the 20 service descriptions (80%) indicated a multidisciplinary team approach. Six of the eleven services that offered reconstruction (55%) also provided psychosexual therapy. The three clinics that did not provide multidisciplinary support (Pennsylvania, Foldes and Manero clinics) all acknowledged this to be an omission. The Manero clinic in Spain was reported to advise women to access sexual therapy 6 months post-operatively (79) and both the Pennsylvania, USA clinic and Foldes service in France were planning to introduce multidisciplinary care (60, 63). None of the four UK services reported offering psychosexual counselling but three provided trauma therapy. Six services (30%) provided trauma therapy only, 5/20 (25%) provided both sexual and trauma therapy, three (15%) provided sexual therapy only and six (30%) did not report the provision of any counselling.

Seven services reported the use of professional interpreters or health advocates to translate. The paper reporting the Birmingham service (UK) mentioned the use of interpreters but it was not clear whether these were specialist interpreters co-located within the FGM/C specialist clinic or the usual interpreting services employed in their maternity setting (69). One paper described the employment of Somali and Arabic-speaking health advocates who act as a bridge between patients and staff (10) and another paper described the use of “cultural mediators” (71). Only 4/20 (20%) services provided details of community engagement work (the HUG, Sunflower, CeMaVIE and IRCCS clinics). Other staff members included forensic science experts, violence against women experts, anthropologists, sexologists, psychiatrists, psychologists, “curators” (undefined), child protection advisors, nurse, case manager, psychosomatic specialist and specialist NGO social workers.

#### 3.3.2 Secondary features: (a) the context of care

Table 5 presents features of the context of care. The first column addresses eligibility. Two services (HUG, Swiss clinic and Akhavan, Sweden, services) describe care for both pregnant and non-pregnant women (of which the HUG clinic describes two distinct care pathways), however Akhavan clarifies that the majority of women were pregnant (58). Five were dedicated maternity services; The Sunflower clinic, UK, was the only service dedicated to non-pregnant women. Eleven services did not specify eligibility. However, it can be assumed that these were for non-pregnant women as they all offered reconstruction surgery.

A second column refers to the referral pathway. Five services did not report this. All five maternity services stated that women are asked about FGM/C at the booking appointment. The Sunflower, Foldes, Netherlands and Dexeus services all accepted self-referrals. Ziyada clarifies that some hospitals in Norway allow self-referral (76). In contrast Karim states that, in Sweden, sometimes gynaecologists refuse to refer women (65).

Services in the UK, France, Belgium, and Australia reported that care was free at the point of delivery and funded by public health. The Dexeus hospital in Spain reported charitable funding, and care in both Switzerland and the Netherlands was recovered by health insurance. Two papers specifically acknowledged that charges may prevent women from accessing care (65, 67). In Norway it was noted that patients pay a subsidized consultation fee although “*some centres had waived this*” (76).

Twelve papers made some reference to theories underpinning FGM/C specialist care. The most common theoretical concepts mentioned were cultural sensitivity/competence, holistic, trauma-informed, health literacy, multidisciplinary, and person-centred care. Papers by Ziyada, Beltran and Jordal discussed theories relating to FGM/C care in more detail (59, 64, 76).

Other factors of interest, extrapolated from the service descriptions, included the location of services. Most services in this review were hospital based, mostly in outpatient departments. The Sunflower clinic (10) was initially community-based, located in a General Practice surgery with the goal being to provide a setting less intimidating than a hospital. However, due to commissioning problems the service was re-located into the hospital. The INSIGHT maternity pathway was the only service to describe community midwives conducting home antenatal appointments (61).

Two papers mentioned the importance of suitable/unlimited time for appointments affecting the quality of received care (54, 75) and one paper described case managers who encourage patients to contact them outside of scheduled appointments (65). Commissioning arrangements were not specifically reported in any of the included papers, but some information could be extrapolated from the publications. The majority of services were publicly available (therefore free at the point of delivery) or refunded via health insurance. Those that were located within private hospitals reported charitable funding and two services reported plans to offer multidisciplinary care, implying expectations of increased resource allocation.

#### 3.3.3 Secondary features: (b) the content of care

Supplementary File S6 reports the content of care. In general, models of care and outcome measures varied considerably, however, information and education provision were strikingly similar. Models of care were heterogenous, but all offered an expert clinician's FGM/C Type diagnosis and most offered deinfibulation for women with Type 3. Fourteen (70%) offered some type of psychological intervention (either trauma therapy or psychosexual). Frequently occurring education or information themes included explanations around female genital and clitoral anatomy, physiology, and sexual function; use of mirrors, pictures and models; discussions about the health risks of FGM/C, legal aspects, and pre- and post-surgery counselling. Outcome measures included a range of patient reported outcome measures, and various validated assessment tools such as the Hospital Anxiety and Depression Scale (HADS), Brief Index of Sexual Functioning for Women (BISFW) and the Female Genital Self-Image Scale (FGSIS). General obstetric and neonatal audits also included information on numbers of women with FGM, FGM type, country of origin, age when suffered FGM/C, number of deinfibulation, timing of deinfibulation etc.

### 3.4 Summary of Key features

Information from earlier tables or the publications themselves, is summarized in Table 6 to illustrate the most important primary and secondary features of specialist services. The first column presents the four main interventions of: deinfibulation, reconstruction, sexual therapy and trauma therapy as described by Johansen et al. (26). Only the HUG service provided every feature of specialist care. Overall, community engagement was the least commonly reported feature. All six maternity services reported undertaking safeguarding assessments, yet only two papers (10, 50) describing care for non-pregnant women specifically mention this.

## 4 Discussion

One of the important premises that this paper was based upon, is that most of the current research into FGM/C care explores the experiences of women within “general” (i.e., non-specialist) healthcare settings (15–24). “Generalist” care for FGM/C survivors might be a General Practitioner appointment where FGM is discussed for the first time, standard care during pregnancy or childbirth, or a general gynaecological consultation. Our aim was to specifically examine the key features of specialist FGM/C care.

The review findings suggest that, currently, there is considerable variability in specialist care models and no established consensus for what constitutes an FGM/C specialist service. In the discussion below, we propose a framework with which to examine the definition and understanding of specialist services, by identifying the primary features found in this review. Secondary features will then be discussed. The final section positions the evidence within the limited context of a scoping review and makes some recommendations for future investigation.

### 4.1 Definition and understanding of specialist services

A key purpose of scoping reviews is to clarify key concepts or definitions in the literature on a particular topic (35, 84). The first challenge was to define what is meant by “specialist” provision as FGM/C care is a complex intervention made up of several different components (85, 86). A search of the wider literature revealed several papers discussing the distinguishing features of specialist vs. generalist care (38, 40–44). From these publications four key/primary features were selected as the most important domains for FGM/C specialist care.

### 4.2 Primary features

#### 4.2.1 Identification as a specialist service

A variety of different phrases were used in the included publications, reflecting the heterogenous configurations of specialist services. The majority named themselves as “specialist clinics” but these did not appear to provide more comprehensive care (in terms of number and variety of support/treatment options) than those services that were called care pathways. It would be interesting to compare the care of women attending an FGM/C clinic (where specialists are co-located on one site) to those attending a specialist care pathway, to see whether and how their care differs.

Although it might be assumed that FGM/C specialist services only provide care for women with FGM/C, this review has identified examples where FGM/C patients were not the sole recipients of care, such as Foldes' (63) general Urology service. Similarly, several papers described care for a small number of FGM/C patients as part of a general plastic surgery, urology, or gynaecology service. These papers were however, included at full text review because they described a specialist FGM/C surgical treatment intervention and an intention to provide multidisciplinary care. However, one could argue that a service that is not solely dedicated to women with FGM/C should not be classed as an FGM/C specialist service and is an example of general care.

#### 4.2.2 Identified expert lead

Another key feature is that the service is led by a specialist, being “*a person who is highly skilled in a specific and restricted field*” (45). In the field of FGM/C there appears to be a variety of different healthcare professionals fulfilling this role. The thirteen clinical leads reported here were either Midwives, Gynaecologists, Urologists or Plastic Surgeons. In the wider literature, Baillot et al. highlighted the fact that often an FGM/C specialist service is set up by an “*enthusiastic committed midwife, obstetrician or gynaecologist ….resulting in the services (becoming) very dependent on the clinician leading them*” (27) and Dawson similarly mentions the expert characteristics of the lead clinician being a key factor in enabling successful and sustainable services (33).

The Royal College of Obstetricians and Gynaecologists (RCOG) 2015 Green Top guidelines state that specialist multidisciplinary FGM/C services should be led by a consultant obstetrician and/or gynaecologist and that all women should be offered referral for psychological assessment and treatment, testing for HIV, hepatitis B and C and sexual health screening (3). We found several instances of services led by midwives or medical consultants from other disciplines. It is not known how or whether clinical leads belonging to different medical professions impact upon the care provision. Perhaps as a minimum, the clinical lead expert should be able to diagnose FGM/C Types, carry out a safeguarding assessment and provide health information and education as part of a commitment to end the continuation of FGM/C (3, 4). However, the Dahlia Project is an example of an FGM/C specialist service which only offers psychological support. Notably, the clinic does not have a “medically” trained clinical lead, therefore, women need referring on to a specialist clinic for FGM/C type diagnosis.

#### 4.2.3 Provision of at least one specialist intervention

A key feature of specialist services is to provide specialist interventions. We used Johansen et al.'s seminal paper (26) to highlight the four main treatment options offered during FGM/C care namely:- deinfibulation, reconstruction, psychological trauma counselling and psychosexual counselling. Like them, we found that reconstruction surgery was only available in certain European countries (either at not cost or via the private/charity sector) and significant regional variation regarding the provision of psychological and/or sexual counselling. The majority of services provided a minimum of at least one surgical (i.e., reconstruction and/or deinfibulation) and one psychological (i.e., trauma and/or sexual counselling) intervention. Although one fifth of services provided both sexual and trauma psychological support, one third (6/20) did not provide counselling at all. This is despite overwhelming evidence of the importance of psychological support for FGM/C survivors (4, 25, 87).

Amongst the papers describing care available to non-pregnant women, all but one were focused upon reconstruction surgery. Reconstruction surgery [which was first introduced to Europe in France in 1998 (88)] is currently not available in all high-income countries, however, given that the surgical techniques and expertise exist to perform gender reassignment surgery, female cosmetic surgery and post-vulvar cancer surgery, this is perhaps a surprising finding. We suggest that one reason for the variability in current reconstruction service provision lies in a tension between evidence and innovation, with healthcare guidance and commissioning bodies in different countries reaching differing conclusion.

Currently, reconstruction surgery is not recommended in the 2016 “WHO Guidelines on the Management of Health Complications from female genital mutilation” under section 3.3.2. as there *“is not yet sufficient evidence of benefit*” (25). However, they do state that “*“Available evidence indicates that reconstructive clitoral surgery can improve chronic clitoral pain as well as dyspareunia symptoms among women who have had clitoral tissue excised or damaged due to FGM.”* It is also noteworthy that a previous section of the guidelines, 3.1.2, recommends deinfibulation surgery, despite stating that there are similar significant gaps in the research surrounding this procedure (such as:- providers not being well informed about how and when to deinfibulate women, particularly during childbirth; a lack of research to understand factors that promote or act as barriers to the uptake of deinfibulation; and limited evidence surrounding the urological consequences (25)). The requirement for evidence underpinning these statements of recommendation seem contradictory yet may have influenced whether some countries provide certain procedures.

In the UK, the RCOG FGM Green Top guidelines state that “*Clitoral reconstruction should not be performed because current evidence suggests unacceptable complication rates without conclusive evidence of benefit*” (3). However they recommend future research includes clinical trials to investigate the safety and effectiveness of clitoral reconstruction (3). They also mention in this guideline that female genital cosmetic surgery (FGCS) may be prohibited unless it is necessary for the patient's physical or mental health. This same argument could be used to advocate for reconstruction surgery for FGM/C survivors (3).

Prior to the WHO guideline recommendation, the issue of reconstruction became highly politicised in the UK. Prominent UK specialists published a commentary in response to a Lancet publication in 2012 describing a pioneering FGM/C reconstruction service, stating (63, 89) “*The report by Pierre Foldès and colleagues claims to show that clitoral surgery after female genital mutilation (FGM) can reduce pain and restore pleasure. However, the claims are not anatomically possible and not supported by current evidence of the effects of clitoral surgery”* (89)*.* This is significant because one of these specialists is co-author of the 2015 RCOG guidelines and is described under author information as a “*founder member of the Female Genital Mutilation Clinical Group, a member of the RCOG FGM Task Force and has provided ad hoc, unpaid clinical and strategic advice on FGM to the Department of Health, Home Office, Director of Public Prosecutions, Health Education England, NHS London and NHS England”* (3). This is an example of how professional positioning, and social & political contexts are interlinked (82).

NHS England's document “Commissioning services to meet the needs of women and girls with FGM”, states “*Until clinical evidence emerges, commissioners are strongly advised not to commission”* (90) *page 5.* However, one might argue that the introduction of surgical interventions such as reconstruction within a robust research programme would allow the evidence base to be built, therefore providing the foundation for change in practice. In fact, the WHO guidelines also state “*a recommendation in favour of this procedure could not be implemented equitably because it is not yet available in the majority of countries with a high prevalence of FGM/C.”* (25) This statement presents a dual challenge: on one hand, the absence of a recommendation primarily due to insufficient research, and secondly, the paradox of not providing the surgery, which if offered, could potentially generate the essential evidence to advocate for the adoption of such a procedure.

A further ambiguity that was noted when reviewing the included papers, was one paper by Akhavan in which reconstruction appears to be confused with deinfibulation (58). We found three other papers in the wider literature, two studies of maternity care in Norway (91, 92) and another describing general maternity care in Sweden (20), which make a similar reference. This illustrates that there may be some confusion about how these types of surgery differ. This not only reveals the complexity around medical terminology and FGM/C care but may reflect the difficulties and awareness required to explain these adequately to women.

#### 4.2.4 Provision of a multidisciplinary team

The majority of services described some form of multidisciplinary care provision. Two services stated their intention to become a multidisciplinary service. This illustrates a common tendency for FGM/C care to evolve over time, from what often starts as a response to unmet need, then expands according to increased demand and eventually results in a decision to establish a dedicated specialist service. Models of specialist care in other disciplines reportedly also often develop iteratively (38) and this appears to be a common feature of expert service development. Although this shows how service commissioning sometimes responds to the demands and needs of the patient population, there is increasing research suggesting that planned, co-designed services are preferable (30, 93).

Because women with FGM/C often present with both physical and psychological symptoms, it is likely that a multidisciplinary service will be optimal. However, in the case of FGM/C, a solely “clinical” multidisciplinary team may not be sufficient. The context within which services operate is crucial, given that FGM/C is a societal, political, and safeguarding issue as well as a health issue. In the Global North, FGM/C survivors may be refugees or asylum seekers. Therefore requiring interpreters, community support workers, support with accessing housing and income, and sometimes input from the wider safeguarding team etc. Furthermore, services should ideally meet the phycological needs of women with FGM/C by providing both trauma and psychosexual therapy. Additionally, there is evidence that mental health provision in tailored to women from the Global North who may have different knowledge and awareness of mental health services than women born or brought up in the Global South and different perspectives/world views that might influence the take up of counselling (17, 27).

### 4.3 Secondary features

Secondary features relate to; (a) the context of care and (b), the content of care. These are particularly important, because the role that education and public health play (alongside clinical professionals), is critical in preventing the continuation of FGM/C.

#### 4.3.1 (a) The context of care

Contextual factors relate to the environment or setting of the healthcare service (46). A UK Royal College of Midwives publication recommends that optimal service configuration takes into account the needs of specific population groups (94). Clients with FGM/C are disproportionately more likely to be refugees or asylum seekers, from minoritized communities (95, 96), and have suffered a form of child sexual abuse with its potential mental health consequences. They are also subject to the constraints of gender inequality. All these factors affect access to care and explain why FGM/C specialist services should aim to reduce barriers such as language, location, and cost. This also demonstrates how health/clinical service provision for FGM has to be supported by wider social and governmental/policy infrastructure.

There is a large body of literature around factors that affect access to care, and several authors have specifically written about this topic and FGM/C (29, 48, 52, 76, 92, 97–99). Notably, even in this small sample of journal articles, three studies, two from the UK (69, 75) and one from Australia (72), report that specialist FGM/C maternity care is often under-utilised (i.e., when women are identified very late in pregnancy), despite the fact that FGM/C should be part of universal enquiry during the antenatal booking appointment (61). Karim notes that relatively small numbers of women have sought a surgical solution to FGM/C in the Netherlands because it is not easily accessible or well-advertised (65) and three other papers mention the impact of publicity/advertising upon attendance to specialist services. Ziyada's paper describing Norwegian healthcare highlights the power differential that exists between women and health professionals and explains how “health literacy” (76) affects access to care. This explains why, as one study remarked (64), women who access FGM/C healthcare often work in the health sector themselves as they have the advantage of knowing how to access services. This has similarly been acknowledged in a recent publication (100).

Four services said they accept self-referrals, and one paper stated that self-referral was allowed sometimes (76). Clearly the absence of strict referral pathways and geographical boundaries encourage easier access to care. Concerningly, one paper from the Netherlands states that sometimes gynaecologists refuse to refer women to specialist services (65). This further illustrates the power differential mentioned in the previous paragraph.

Eight papers (40%) made no reference to theoretical concepts that underpin care and no publication mentioned co-designed care (33, 93). However, there is a growing demand for services in the Global North to move away from the old biomedical models of care and to become more inclusive and responsive to service users needs and demands (101, 102). In the field of FGM, there is a considerable amount of literature examining theories related to behaviour change and FGM prevention (103, 104), but little research has examined the implications that theory has in determining how health care services are delivered, and their success in responding to the health needs of women who have experienced FGC/M (28). For example, two seminal clinical texts produced by the WHO, “Care of women and girls living with female genital mutilation: a clinical handbook” and “WHO guidelines on the management of health complications from female genital mutilation” make no mention of theoretical underpinnings.

Costs ranged from free public health services or reimbursed health insurance to charity funding. Only Norway reported actual payment requirements. However, in the wider literature, one paper from the U.S.A (105). describes the case of a woman who was forced to have deinfibulation under local anaesthetic because she did not have health insurance, which would have afforded the procedure under general anaesthetic. This illustrates the impact funding can have upon specialist care.

One third of studies mentioned the use of specialist interpreters co-located within the service, but few reported community engagement work. Interestingly, a recent scoping review describes the role of facilitators/enablers for FGM/C care access and concludes that these are not well evaluated (33). Furthermore, there is some evidence that hierarchical tensions can arise between “volunteers” “in the community” and the “professionals” who may “supervise” them. Particularly if the former receive little financial support, but the latter have salaries and occupational status (82, 106, 107).

Few papers gave comprehensive information about how their services were commissioned. Such information is potentially important because it is closely linked to the sustainability of services. Throughout the Global North it is likely that areas of low prevalence may not have any FGM/C service. It should also be acknowledged that FGM/C services which have not published data in academic papers are unreported and largely invisible (For example, the Waltham Forest clinic (10) and the work of Dr Abe (82), both in the UK). FGM/C specialist care in the UK has recently been referred to as suboptimal (for example, psychological support is frequently under-resourced) and precarious (likened to a postcode lottery) (27, 106). This is particularly significant if services depend upon the commitment and passion of one person (9, 108) with the implication that, when/if that person moves on, the service may close.

Although our inclusion criteria included adult service provision only, it should be acknowledged that maternity services usually cover women that are under 18 years old. Furthermore, some of the services for non-pregnant women, included in this review, did not specify whether there was an age limit for attendees. The majority of services were located within a hospital setting but some papers specifically mentioned the use of outpatient departments and one paper specifically described a service based within a community-based General Practitioner (GP) health centre with the aim to make this easier to access than hospital-based care.

#### 4.3.2 (b) The content of care

Overall, the information pertaining to models of care was extremely variable. Multi-disciplinary care was the primary model in most papers, typically with a minimum of one surgically trained doctor or specialist midwife lead working alongside a mental health professional. WHO recommend holistic care for women with FGM/C (25), however we found this is not always the case. Noticeably, those services offering reconstruction for non-pregnant women were more likely to be accompanied by psychosexual therapy, whereas services for pregnant women rarely provided any counselling. Ideally holistic (i.e., both trauma and psychosexual) therapeutic support should be available for all women.

Although not every paper discussed education/information provision in detail, those that did, covered similar topics. Information provision is a component of FGM/C care considered by the WHO to be integral to prevention work to end FGM/C (27). It is well documented that one of the reasons why FGM/C has been perpetuated is because women, families and communities were unaware of the damaging physical and psychological consequences of FGM/C. For example, often when attending specialist appointments, women have said that they previously believed that difficulties passing urine or pain during sexual intercourse were the “normal” sufferings of womanhood (10). Accordingly, the WHO defines Information, Education and Communication (IEC) interventions as “a public health approach aiming at changing or reinforcing health-related behaviours” (25).

### 4.4 Pregnant vs. non-pregnant care

This review found clear differences between the care offered to pregnant and non-pregnant women with FGM/C, in keeping with other evidence reviews (31, 32). Interventions reported here which are not included in either Chappel's scoping review (31) or Balogun's Cochrane systematic review (32) were perineal physiotherapy, family planning (54) and paediatric support and examinations (61). Although maternity and gynaecological care for women with FGM/C sometimes differs, we found that safeguarding was rarely reported in the descriptions of services for non-pregnant women. This may be due to clinicians being fearful of deterring women from accessing healthcare (109). It is recognised that every healthcare professional encounter with an FGM/C survivor is an opportunity to prevent FGM/C being perpetuated (110). Therefore, the consequences of not discussing safeguarding are significant and concerning. However, recent evidence from the UK has highlighted the potentially adverse effects of policies that focus on safeguarding, rather than emphasizing the role of education, community enabled prevention work and specialist healthcare support (111). It has been hypothesized that policymakers in the UK (influenced by media rhetoric) failed to appreciate that by far the majority of FGM/C cases in the UK are historic cases; that the real risk to girls may have been overestimated (112, 113); and they failed to acknowledge the “*shifted societal norms around FGM/C” (e.g., FGM/C no longer bestowing social advantage within migrant communities)* (111)*.* Moreover, the threat of a safeguarding intervention may cause some women to feel alienated and stigmatized (114) resulting in fear and reluctance to attend specialist FGM services (115). In fact, there is evidence that safeguarding may negatively impact on women's willingness not only to access health care for FGC/M but also for other health concerns—thus potentially compromising their overall health and well-being (114).

One might even argue that those specialist health services (regarded as safe trauma-informed spaces which focus upon psychological interventions), should discuss safeguarding with a women regarded as a victim of child abuse. Might there be some situations/contexts where safeguarding is not an appropriate intervention?

Furthermore, all healthcare interactions are opportunities to discuss available treatment options, however in this review reconstruction does not appear to be mentioned in relation to services for pregnant women, despite being a topic relevant to the woman's long term reproductive health.

### 4.5 Recommendations and research priorities

Based on these findings, we believe there are several issues which deserve further attention. With regard to primary features of FGM/C specialist care we suggest that all services, regardless of whether or not they provide reconstructive surgery, should: universally provide access to deinfibulation; assess psychosexual needs; and provide psychosexual counselling; in addition to trauma assessment and therapy. In relation to the content of care, we recommend that non-pregnant women who access FGM/C care, should always receive a holistic safeguarding assessment, similarly to that provided for pregnant women. It is essential that the expert lead in a health setting is able to diagnose FGM/C type, undertake a safeguarding assessment and provide health information and education to ensure that FGM/C is no longer perpetuated.

In terms of future research, there are a number of areas which warrant further investigation. We found that services offering reconstruction surgery were more likely to provide psychosexual support, whereas those services which did not provide reconstruction were unlikely to provide this. It would be interesting to determine whether this is due to funding decisions, research priorities or other factors. Furthermore, there appeared to be a distinction between the research produced by those countries which provide reconstruction and those which do not. Notably UK, Norway and Australia, which do not offer reconstruction, published papers which focused upon the care of pregnant women, whereas the countries (in this review) that offer reconstruction (being Spain, Sweden, Switzerland, Italy, France, Belgium, Netherlands, USA) rarely mentioned the care of pregnant women. Future research could examine this discrepancy.

Further research could also compare the distinction between, and experiences of, services for pregnant women attending an FGM/C clinic (with a co-located multidisciplinary team) to those attending a specialist care pathway. In addition, studies are needed on the outcomes of surgical and non-surgical treatment options offered within specialist services (25).

This scoping review has shown great variability in specialist service definition and specialist service provision. In order to inform guidelines and commissioning decisions for FGM/C care in the future, two new areas of research are required: (1) exploring what key stakeholders feel should be the optimal model of care and what their experiences are of the current models; and (2) given the notable ambiguity and variability of practice around reconstruction surgery, to review the evidence on reconstruction. This could involve finding out what stakeholders want regarding reconstruction and generating high quality evidence regarding service models and outcomes of reconstruction, with which to inform future health service decision making. It would also be interesting to investigate the influence of WHO guidelines upon access to surgical interventions in those countries where it is not offered, as well as what other influences may be at play.

### 4.6 Strengths and limitations

It is well known that care provided to women with FGM/C within “general” healthcare settings is sub-optimal (28, 30, 48, 116, 117). There is little evidence or understanding however of what an FGM/C specialist service should look like. This scoping review sought to address this gap and is the first of its kind to describe the features of FGM/C specialist services. As such, its findings make a unique contribution to the field.

The review presented a number of challenges, however. The first relates to the conceptualisation and reporting of “specialist” services. Only 25 papers over a 10-year period provided any description of the model of FGM/C care. Although these papers included studies from several different countries and represented a wide range of research methods, the level of detail about participants, context and content was extremely variable. Some papers with very limited information were excluded at the screening stage, which means that their services are not represented in this review. Others provided heterogenous information, making it challenging to compare services. There were several examples where two descriptions of one service were unclear or conflicting. Overall, a potential limitation is that some services or features of care may have been missed due to lack of reporting. We should also acknowledge that there may be many specialist FGM/C services across the Global North that deliver excellent care but have never been reported upon in the academic literature.

The authors acknowledge that the literature search was restricted to primary research sources due to time limitations. Only one textbook source was identified which included clinical services as a discussion item (82). This is a review of clinical service provision, therefore the constraint of considering only clinical practice via “scientific” academic papers results in omitting a substantive review of textbooks, media coverage and information contained in other sources.

In terms of review methods, a single reviewer undertook the primary screening, data extraction and merging of papers, which may have introduced a degree of selection bias. JA is a specialist FGM/C service lead in the UK which may have influenced the choice of papers and identification of key features included in this review. However, JA's in-depth knowledge of the field was also a key strength in terms of interpreting the sometimes unclear or contradictory data and sourcing relevant papers through supplementary approaches.

## 5 Conclusion

Based on the analysis of service characteristics, this review has identified and described the components of current FGM/C specialist service provision in the Global North. Included studies demonstrated the existence of multiple configurations of FGM/C specialist care provision.

First we defined the components of “specialist” (as opposed to “generalist”) care and then applied these to an analysis of FGM/C specialist care. FGM/C specialist provision was categorised into primary (essential for identification as a specialist service) and secondary (all other) features. Primary features were:—(i) described as “specialist”; (ii) with an expert lead (typically a surgically trained doctor or specialist midwife/nurse); (iii) a multidisciplinary team (usually providing a counsellor alongside the clinical lead); and (iv) offering a minimum of one psychological or one surgical intervention. Secondary features included (a) the context and (b) the content of care, including factors affecting access to services, such as cost, eligibility/referral pathways, advertising, availability of interpreters, community engagement, education, and theoretical underpinning.

Overall, we found that services vary, both between and within countries, and the absence of established evidence-based criteria for high quality services potentially impacts upon the quality and standards of care received. Principles of “Respectful Maternity Care” (RMC) promote themes of equitable access to evidence-based care globally (118). As FGM/C affects 5% of the global female population, FGM/C specialist care should be available across all settings and standards should be established to ensure consistent quality.

We found that only the HUG service in Switzerland demonstrated all key features of specialist care. The variability of services identified in the review highlights the fact that women's access to appropriate specialist care varies depending upon where they live, leading to inequalities. The variability was particularly notable in 3 areas: (i) women in some high-income countries were unable to freely access reconstruction surgery with psychosexual support; (ii) mental health support was rarely integrated into pregnant women's services; (iii) information/education and safeguarding assessments may not be universally embedded in care provision or maybe under-reported.

WHO guidelines currently recommend access to deinfibulation, mental health support, sexual counselling, and education and information. However, these are not always available, and there appears to be no clear pattern as to the features of FGM/C specialist services, treatment options and staffing configurations. Given the variability in care access and provision demonstrated in this review, we suggest further research is needed to elucidate optimal care models and pathways and to develop a comprehensive framework for evaluating FGM/C clinical specialist care.

The conceptualisation of primary and secondary specialist care features developed for this review can form a useful framework to guide future research and service development in this area. This is needed to enable the examination, comparison, and assessment of clinical features to determine their effectiveness in delivering positive outcomes. Indeed, currently a geographical lottery appears to exist, not only within the UK, but also across the Global North.

## Data Availability

The original contributions presented in the study are included in the article/**Supplementary Material**, further inquiries can be directed to the corresponding author.

## Supplementary material

Supplementary File S1

Prisma Checklist.

Supplementary File S2

Search Terms.

Supplementary File S3

Search Strategy.

Supplementary File S4

Reason for exclusion.

Supplementary File S5

Data Extraction template.

Supplementary File S6

The Content of Care.
