# Supplementary material for: Examining the key features of specialist health service provision for women with Female Genital Mutilation/Cutting (FGM/C) in the Global North: a scoping review
Source: Front Glob Womens Health. 2024 May 22;5:1329819. doi: 10.3389/fgwh.2024.1329819 (PMC11150566; doi:10.3389/fgwh.2024.1329819)
Supplement: Supplementary file 1 [file Table1.docx]

Supplementary File 2

## Search Terms

| Key Search Terms |  |
| --- | --- |
| Participants | Female genital mutilation; Female circumcision; female genital cutting; removal female external genitalia, clitoris removal / injury, clitoridectomy.FGM, FGC, excision, infibulation, ritual cutting, ritual circumcision, pharaonic |
| Concept | service configuration, theoretical underpinnings, treatments/therapy/interventions, models of care, surgery/procedures, reconstruction, follow up measures/outcomes; (integrated) care pathway, referral pathway, organisation of care, commissioning/setting up services, guidance, protocols, specialist services; healthcare delivery; Healthcare/health-care/health care, health, service(s), model(s), clinic(s), clinical practice, community/community-based, holistic, integrated, multi-disciplinary, medical, surgical, psychological, therapeutic, configuration, guidelines, specialist; referral, commissioning, access, psychosocial, intervention reconstruction, deinfibulation/defibulation/reversal, women-centred, person-centred care, hospital |
| Context | Organisation for Economic Co-operation and Development (OECD), high incomes countries, Europe, Australia, Austria, Belgium, Canada, Chile, Colombia, Costa Rica, Czech Republic, Denmark, Estonia, Finland, France, Germany, Greece, Hungary, Iceland, Ireland, Israel, Italy, Japan, Korea, Latvia, Lithuania, Luxembourg, Mexico, Netherlands, New Zealand, Norway, Poland, Portugal, Slovak republic, Slovenia, Spain, Sweden, Switzerland, Turkey, United Kingdom, United States, Europe, Scandinavia, England, Wales, Scotland, Ireland, Northern Ireland. |
