# Supplementary material for: Examining the key features of specialist health service provision for women with Female Genital Mutilation/Cutting (FGM/C) in the Global North: a scoping review
Source: Front Glob Womens Health. 2024 May 22;5:1329819. doi: 10.3389/fgwh.2024.1329819 (PMC11150566; doi:10.3389/fgwh.2024.1329819)
Supplement: Supplementary file 2 [file Table2.docx]

## Supplementary File 3 - Search Strategy

Ovid MEDLINE(R) ALL <1946 to November 02, 2022>

| **#** | **Query** | **Results from 3 Nov 2022** |
| --- | --- | --- |
| 1 | female genital mutilation.ti,ab. | 1,330 |
| 2 | female genital cutting.ti,ab. | 300 |
| 3 | female circumcision.ti,ab. | 432 |
| 4 | FGM.ti,ab. | 1,540 |
| 5 | FGC.ti,ab. | 480 |
| 6 | (excision adj4 (wom?n or female* or girl*)).ti,ab. | 784 |
| 7 | (excision adj4 (genital* or clitor* or labia*)).ti,ab. | 151 |
| 8 | infibulation.ti,ab. | 175 |
| 9 | (ritual cutting adj (wom?n or female* or girl*)).ti,ab. | 0 |
| 10 | (ritual circumcision adj4 (wom?n or female* or girl*)).ti,ab. | 2 |
| 11 | pharaonic circumcision.ti,ab. | 17 |
| 12 | (sunna.mp. or sunnah circumcision.ti,ab.) adj4 (wom?n or female* or girl*).mp. [mp=title, book title, abstract, original title, name of substance word, subject heading word, floating sub-heading word, keyword heading word, organism supplementary concept word, protocol supplementary concept word, rare disease supplementary concept word, unique identifier, synonyms] | 9 |
| 13 | clitoridectom*.ti,ab. | 98 |
| 14 | 1 or 2 or 3 or 4 or 5 or 6 or 7 or 8 or 9 or 10 or 11 or 12 or 13 | 3,981 |
| 15 | (healthcare or health-care or health care or health).ti,ab. | 2,490,440 |
| 16 | (service* or model* or clinic*).ti,ab. | 8,265,647 |
| 17 | clinical practice.ti,ab. | 232,722 |
| 18 | (community or community-based).ti,ab. | 591,378 |
| 19 | holistic.ti,ab. | 31,935 |
| 20 | integrated.ti,ab. | 300,360 |
| 21 | (multi-disciplinary or multidisciplinary or interdisciplinary).ti,ab. | 157,950 |
| 22 | (provision or provider).ti,ab. | 161,451 |
| 23 | (medical or surgical or psychological).ti,ab. | 2,555,273 |
| 24 | (therapy or therapeutic).ti,ab. | 2,988,173 |
| 25 | treatment.ti,ab. | 4,980,083 |
| 26 | configuration*.ti,ab. | 158,755 |
| 27 | guideline*.ti,ab. | 441,632 |
| 28 | protocol*.ti,ab. | 546,405 |
| 29 | framework.ti,ab. | 336,826 |
| 30 | (theor* or theoretical).ti,ab. | 768,015 |
| 31 | speciali*.ti,ab. | 270,161 |
| 32 | referral*.ti,ab. | 135,722 |
| 33 | commission*.ti,ab. | 47,676 |
| 34 | access*.ti,ab. | 639,378 |
| 35 | intervention*.ti,ab. | 1,239,012 |
| 36 | reconstructi*.ti,ab. | 286,852 |
| 37 | care.ti,ab. | 1,682,046 |
| 38 | (deinfibulation or defibulation or reversal).ti,ab. | 77,550 |
| 39 | (wom?n centred or wom?n-centred or person centred or person-centred or patient centred or patient-centred).ti,ab. | 11,224 |
| 40 | hospital.ti,ab. | 1,175,732 |
| 41 | 15 or 16 or 17 or 18 or 19 or 20 or 21 or 22 or 23 or 24 or 25 or 26 or 27 or 28 or 29 or 30 or 31 or 32 or 33 or 34 or 35 or 36 or 37 or 38 or 39 or 40 | 16,437,147 |
| 42 | exp canada/ or exp mexico/ or exp united states/ or chile/ or Costa Rica/ or Colombia/ or israel/ or turkey/ or exp japan/ or exp Republic of Korea/ or austria/ or belgium/ or exp baltic states/ or estonia/ or latvia/ or lithuania/ or czech republic/ or hungary/ or poland/ or slovakia/ or slovenia/ or exp france/ or exp germany/ or united kingdom/ or exp england/ or northern ireland/ or exp scotland/ or wales/ or greece/ or exp ireland/ or exp italy/ or luxembourg/ or netherlands/ or portugal/ or exp "scandinavian and nordic countries"/ or exp denmark/ or finland/ or iceland/ or norway/ or sweden/ or spain/ or switzerland/ or exp australia/ or exp new zealand/ or (Australia or Austria or Belgium or Canada or Chile or Colombia or "Costa Rica" or "Czech Republic" or Denmark or Estonia or Finland or France or Germany or Greece or Hungary or Iceland or Israel or Italy or Japan or "Republic of Korea" or "South Korea" or Latvia or Lithuania or Luxembourg or Mexico or Netherlands or New Zealand or Norway or Poland or Portugal or "Slovak Republic" or Slovenia or Spain or Sweden or Switzerland or Turkey or "United Kingdom" or England or Ireland or Scotland or Wales or "United States").ti,ab. | 3,951,309 |
| 43 | europe*.ti,ab. | 338,391 |
| 44 | (high-income or higher-income or high income or higher income).ti,ab. | 20,834 |
| 45 | 42 or 43 or 44 | 4,167,264 |
| 46 | 14 and 41 and 45 | 628 |
| 47 | limit 46 to yr="2012 - 2022" | 390 |

https://ovidsp.ovid.com/ovidweb.cgi?T=JS&NEWS=N&PAGE=main&SHAREDSEARCHID=3BJ54qevGX4ClxgEX521bJai7BHFK8d1EAow20gFxnXoWSZc4PJJZuYEi0VFb2
