# Supplementary material for: Examining the key features of specialist health service provision for women with Female Genital Mutilation/Cutting (FGM/C) in the Global North: a scoping review
Source: Front Glob Womens Health. 2024 May 22;5:1329819. doi: 10.3389/fgwh.2024.1329819 (PMC11150566; doi:10.3389/fgwh.2024.1329819)
Supplement: Supplementary file 3 [file Table4.docx]

## Supplementary File 6 – The Content of Care

|  | **Model of Care** | **Outcome measures** | **Infomation / Education given to women** |
| --- | --- | --- | --- |
| HUG maternity clinic, Geneva #1a | Prenatal consultation - women examined and counselling and recommendations regarding defibulation if Type 3. Discuss low threshold for performing an episiotomy, and prevention of FGM/C for neonate. Follow-up appointments scheduled after delivery. Women choose if prefer partial deinfibulation or total opening. Women who request reinfibulation given long-term follow-up, including counselling and post-partum perineal physiotherapy. | Type of delivery, reason for caesarean delivery or assisted delivery, blood loss, episiotomy, perineal tear, duration of 2nd stage of labour, postpartum complications, weight of neonate, Apgar score. | Information about female anatomy, physiology, and sexuality. With patient’s consent, husband included in discussion on the advantages of not re-stitching for micturition, menstruation, sexual intercourse, future deliveries, or eventual gynecological investigations. |
| HUG gynaecology clinic, Geneva #1b | Women requesting clitoral reconstruction undergo psychosexual evaluation and therapy with psychiatrist or psychologist, (both trained sex therapists). 3 sessions exploring psychological, biological, pharmacological, relational, and contextual factors affecting sexual response. Sex therapy (8 sessions before & 4 sessions after surgery) consists of counseling and psychotherapy and address specific causes of sexual dysfunction. (e.g. past or present psychiatric conditions, sexual pain, desire, arousal, pleasure, orgasm, autoeroticism, relationship factors, past experiences (including the FGM/C), and body image). Postoperative expectations and reasons for requesting CR are discussed | Follow up at 1 week and 1, 3, 6, and 12 months post-surgery  Histopathological analysis  socio-demographic, clinical,surgical variables | Information about sexuality  Health education. Detailed explanations on female anatomy and physiology in women with and without FGM/C;  Detailed information about surgical technique, its outcomes, and current lack of conclusive evidence.  Use a mirror to explain type of FGM/C. |
| Akhavan #2 | NR | NR | NR |
| Sunflower clinic, #3 | Trauma counsellor & Somali & Arabic-speaking health advocates co-located in clinic with specialist midwife lead. Integrated into consultations (with woman’s consent). Counsellor and health advocates offer support during deinfibulation. Health advocates translate during 1-1 counselling session. Offer walk-in same day deinfibulation under local anaesthetic. Fast tracked for referral to linked consultant for deinfibulation under general anaesthetic. Women seen within 2 weeks of referral.  Safeguarding assessment for all women. Medical reports for asylum seekers. Up to 8 initial counselling session but may be more if required. | Regular audit – Socio-demographics; Number of deifibulations. Follow up appointment two weeks after procedure. Evaluation form filled.  Hospital Anxiety and Depression Scale (HADS) and Generalized Anxiety Disorder 7 (GAD7) | Before surgery, woman receives detailed information about female anatomy, physiology, and deinfibulataion procedure. Physical and psychological health consequences of FGM/C type of FGM/C explained using line drawings. Use of mirror if woman wants. Discussion around UK law, FGM/C is human rights violation and form of child sexual abuse, woman’s right to bodily integrity and sexual pleasure, difference between FGM/C and male circumcision. Barnardo’s world map used to show prevalence and law in woman’s country of origin. |
| Bicetre, #4 | Inform patient about type of excision she has undergone, reassure her about consequences on her sex life and motherhood. Help her locate her clitoris and possibility of stimulating it. Psychological consultation to assess & treat post-traumatic sequelae related to excision or other trauma. Sexological consultation to evaluate & possibly change sexual satisfaction. Multidisciplinary meeting, share information and reflections to try to find the most appropriate response for patient. | NR | Explain that external part of clitoris is cut while greater part of organ is internal, always present under skin & can be stimulated. Describe excitement or orgasmic discharge. Show the genitals (using a mirror). Perineal exercises, sensory focus & different advice regarding sexual mechanics. |
| CeMaViE, #5 | 5 consultations are mandatory before surgery. Expert midwife identifies demands and expectations related to care and surgery. She collects personal and medical history and identifies any gender-based violence & reassures patient about clinical exam. Midwife links with specialized NGOs and social workers. Gynaecology Surgeon examines patient to see if operation is feasible, explains surgical procedure, potential complications and how long recovery period can take. Appointments with psychologist and sexologist next. Psychologist evaluates demand for reconstructive surgery and provides therapeutic management of psychotrauma if needed Eye Movement Desensitisation and Reprocessing method is used, a cognitive behavioural therapy.  Sexology consultation covers: (a) anatomy, (b) the impact of FGM/C on sexual response (c) reality of female sexual response, (d) false beliefs (e) sexuality as an active learning process.  Team discusses surgery requests in monthly staff meeting. If decision of meeting is favourable, practical details are reviewed again with patient & gynaecologist at 5^th^ consultation to ensure full understanding of the procedure and postoperative care. The practical aspects of surgery are explained, and intervention scheduled. Mdt follow up: - 3 weeks.  2 months; 6 months; 12 months | Data on self-esteem and empowerment are collected by an anthropologist specialized in FGM/C. Quantitative data collected using validated questionnaires: Hospital Anxiety and Depression (HAD) scale, Brief Index of Sexual Functioning for Women (BISFW), and Female Genital Self-Image Scale (FGSIS) | Drawings of FGM/C types & 3D clitoris used to inform the patient about anatomy of clitoris and impact of FGM/C. Mirrors used if woman wants. Raise awareness that all vulvas are anatomically different. Patient is given evidence-based information based on most recent findings in scientific literature. |
| Pennsylvania clinic, #6 | Preoperative assessment. Late 2020, organized and implemented an FGM/C Centre at our institution. The centre includes sex therapists, psychologists, gynaecologists, and uro-gynaecologists as key partners in pre- and postoperative care. Average follow up was 305 days. | Clitoral, labial, and donor site surgical site occurrences (SSO) & need for revision operations. Patient-reported outcomes using adapted version of Female Sexual Function Index (FSFI). | We support surgical technique with patient education and compliance with postoperative regimens |
| INSIGHT, Scotland, #7 | Primary visit offered in-home. FGM/C management planning, investigation into family’s views on FGM/C, intentions for their children. Offer support with language assistance, employment, informal counselling & links to local community organisations. Referral for examination to Insight obstetrician. Primiparous women and those with Type-III FGM/C may receive obstetric referral to ensure informed decisions regarding their birth plan and potential for intrapartum intervention. At 20 weeks Midwife carries out a risk assessment. May refer woman to Insight Child Protection Advisor if required. | NR | Education on FGM/C and Scottish law |
| Nantes Teaching hospital, #8 | Sexological consultation as part of a multidisciplinary approach. Preoperative stage, sexologist helps woman to put into words her suffering, her experience of circumcision and to explicitly formulate what it is she is seeking, all the while respecting her temporality; Sexologist evaluates woman’s sexual health | Questionnaires evaluate quality of patients’ sex life before and after. 19 items scored on a scale of sexual satisfaction ranging from 0 to 10, components. + Female Sexual Function Index (FSFI) questionnaire about clitoral sensations, symptoms of depression or anxiety, and self-esteem before; 3 & 6 months after the surgery. | Sexologist provides information, sex education and cognitive reframing with the aid of simple pedagogic tools and based on a cognitive behavioural approach. |
| Foldes clinic, #9 | Patients fill questionnaire at entry about their characteristics (age, country of origin, country of excision) and preoperative clitoral pain and clitoral pleasure. We assessed the patients’ expectations for pain and clitoral pleasure on 5-point scales. Clitoral reconstruction performed by one Surgeon under general anaesthetic. Patients discharged within 2 days of surgery. “Weeks after surgery, they are examined and asked to come back in a year’s time. We inform them that postoperative pain would last for about 2 weeks & the wound takes 2 months to heal (epithelialisation), at which point they can resume sexual intercourse. At 1-year visit, women questioned about pain and functionality | Patients filled out a non-validated rating scales for clitoral pleasure and pain on 5-point scales. These scales pragmatically described the patients’ sensations. | Patients were informed orally and in written form about efficiency and side-effects of surgical procedure |
| Karolinska University Hospital, #10 | Women meet a psychosexual therapist in order to be eligible for surgery. They talk about their psychosexual and FGM/C related problems, and fill in questionnaires about their sexual and psychological state | Pre-operative and follow up interview | They learn that not the entire clitoris had been removed during the cutting and explained that this is also the basis for Clitoral Reconstruction surgery, since it brings to the surface underlying clitoral tissue and ‘puts it in its right place’ At the post-operative check-up they compare pre- and post-operative genital pictures |
| Amsterdam #11 | Pre-operative interview by a team: - case manager, female nurse, & surgeon. Patients completed a questionnaire to record demographics. Patients were asked to select a specific, primary motive for requesting surgery. Postoperatively, all patients received 500/125 g oral amoxicillin/clavulanic acid, three times daily, for 5 days. Women that underwent only clitoris reconstructions were discharged on day of surgery, with instructions to maintain low activity for 3 days. Women that required labial reconstruction remained in hospital for 24 h under close observation in case of wound complications. Then, they were discharged and advised to maintain low or minimal activity for 5 days. Patients were advised to allow 3-week recovery period, where they should limit activities, cool the wound, avoid smoking, and avoid heavy physical chores. They were advised full wound healing could require 6 to 12 weeks. Routine follow-ups were conducted in outpatient clinic at 3 weeks, 3 months, 6 months, and 1 year after surgery. Case managers encouraged all patients to contact them outside of scheduled appointments, whenever necessary. | After surgery, postoperative outcomes were evaluated by use of structured questionnaires. Patients were asked to select between (i) satisfaction or (ii) dissatisfaction with the surgical outcome. Additionally, the preoperative expectation was compared to the postoperative satisfaction for each patient to determine whether the main reason for surgery was resolved. | Patients were informed about the possible outcomes, limitations, and possible complications of the surgical procedure |
| Manero clinic, # 12 | Preoperative assessment includes genital examination and collection of sociodemographic characteristics. Patients complete two questionnaires. Surgery is performed as an outpatient procedure. Postoperative care includes bedrest for 5 days to decrease risk of graft mobilization. Postoperative day 5, urinary catheter is removed, and graft is examined. If it has survived, the wound is left uncovered and patient is trained in postoperative care, including daily washing with soap and water. When there is partial graft necrosis, bacitracin zinc– polymyxin B moisturize ointment is applied every 12 hours. The patient is seen every 24–48 hours until its entire reepithelization. If there is total necrosis of the graft, it is surgically removed and primary closure using the surrounding vulvar skin is performed during the same surgical procedure. Patients are scheduled for outpatient clinic visits 1, 3, 6, and 12 months postoperatively. Patients are encouraged to work with a certified sex therapist starting at 6 months postoperatively. | The Female Sexual Function Index & Female Genital Self-Image Scale before surgery and at the 6-month postoperative visit. Patients are assessed for personal venous thromboembolism risk using the Caprini score. | Patients are advised about postoperative care and the importance of sexual therapy. |
| Dexeus clinic, #13 | Patients were assigned a therapist in the Department of Psychiatry, Psychology and Psychosomatics before surgery and 6 months following surgery in a face-to-face clinical interview by a clinical psychologist. Assessment of sexual dysfunction, depression levels and sexual distress in Type I and II FGM/C patients prior to a combined intervention involving clitoral reconstruction and psychoeducational intervention, and at a 6- month follow-up.  Patients discharged one day after the surgery, and check-up visits scheduled at 2 weeks, a month and a half, and 3 months after surgery. Patient is instructed on how to perform daily wound care the first month following surgery. Final evaluation at 6 months. | Beck Depression Inventory  Female Sexual Distress Scale Revised  DSM-5 criteria (Patients’ sexual dysfunction) | Psychoeducational intervention consisted of two 20-min outpatient sessions completed before surgery & at 6-month follow-up. Goal to minimize psychological distress associated with clitoral reconstruction and to manage physical and sexual complications that can appear following surgery. Psychosexual information focused on correcting false myths related to female sexual function and sexual practice & strategies on how to cope with a new viewpoint of their sexuality because of the cultural change stemming from migration. |
| Birmingham Heartlands, #14 | A perineal examination is offered to confirm type of FGM/C. Further interventions that may be needed during labour and birth, such as mediolateral episiotomies or deinfibulation, are discussed. They explain legal implications of FGM/C in UK and ensure those who request reinfibulation post-partum are made aware that it is illegal. For women with type III FGM/C antenatal deinfibulation is encouraged but, if declined, intrapartum deinfibulation is planned. Female infants have presence of normal female genitalia recorded in the notes. Health visitors must be notified of maternal FGM/C prior to community handover. | To audit the service against standards set by the trust. describe obstetric outcomes of women with FGM/C & compare adverse outcomes between women who were deinfibulated prior to labour with those who opt for intrapartum deinfibulation. | Counselling re antenatal or intrapartum deinfibulation |
| IRCCS, Italy #15 | 1st meeting is scheduled according to a multidisciplinary approach, involving the appointed gynaecologist-obstetrician together with a psychologist and a cultural mediator. A psychological workshop, focused on the unique experience of the patient, aims to build a solid and trustworthy relationship with the woman, to detect any possible uneasiness connected to the infibulation or to the migratory process and the related consequences (lack of family affection, integration, cultural and language difficulties, change of habits, economic problems and difficult access to health services). Afterwards procedure’ risks and benefits were listed to the patient, and informed consent was obtained. Post-surgery expectations are to be talked over during the preparatory stage | Patient was discharged home the day after the procedure and reviewed in the clinic on the 8th postoperative day, 2 weeks later and after one month. 6 month follow up appointment | Make the patient aware of type of mutilation she has and subsequent consequences which would affect her health. Information provided (with graphic support) to increase awareness on differences between mutilated and non-mutilated female genital apparatus. Specific bespoke information is provided to patient and benefits of the surgery are explained; To clearly and thoroughly describe the surgery, how and when the “reopening” will take place: patient and the medical staff decide how much time is required, who will be attending the surgery, type of anaesthesia, how long the convalescence will last, what the next steps will be. Information is provided on physiological changes following the surgery (e.g., modification in urinary and menstrual flow, changes in vaginal secretions and possible alterations during sexual intercourse) |
| Perth Hospitals, Australia #16 | Women asked at booking whether have FGM/C. All are referred to social services and have an inspection of genitalia in antepartum period. Given appointment with a senior doctor (either registrar or a consultant) for a labour, birth and postpartum management plan. | Audit | Discuss consequences of FGM/C  And advised of legal consequences of practising FGM/C in Australia. |
| Sydney Metropolitan, Australia #17 | Follow guidelines by the Royal Australian and New Zealand College of Obstetricians and Gynaecologists (RANZCOG). Type of FGM/C is documented at 1st booking visit and a clear care pathway for antenatal care, labour and birth is established and documented for each woman. Counselling, psychological and translation services are provided. FGM/C health risks & legal framework are discussed. Opportunity is created for women and partners to explore their perceptions and beliefs regarding FGM/C. Benefits of deinfibulation are explained and the procedure is offered preferably in the second trimester to allow for vaginal examinations to assess progress in labour. It is, however, also performed during labour. | Rates of maternal and neonatal outcomes for women. | Provide education sessions for migrant women and their partners on FGM/C. |
| UCLH #18 | Questioning at booking and completion of FGM/C section in handheld obstetric notes and computerised maternity information system. Referred to African Women’s Clinic for further assessment. Clinic offers deinfibulation as an outpatient procedure, as well as specialist obstetric and psychological advice. Women with previous FGM/C should have antenatal assessment of anatomy and the need for deinfibulation. All women who do not speak English offered interpretation services. Women with FGM/C assessed by a senior obstetrician when admitted to labour ward. If give birth to a female new-born should be informed that FGM/C is a criminal offence & health visitors should be informed of every woman with FGM/C who has given birth to a female new-born. | General obstetric outcomes collected for audit purposes. | Information on the legal status (HM Government 2011) and health risks of FGM/C must be given to mothers of female babies |
| Norway #19 | Offer deinfibulations & cyst removal.  Psychological and sexual counselling was not part of the offered FGC-specialized services. Long waiting list for appointments left them suffering for several months.  Participants appreciated being offered more than one consultation to discuss their concerns. Some healthcare providers discussed pain management during & after-deinfibulation and presented various pictures of vulva before & after deinfibulation | NR | Gynaecologists explained how deinfibulation would alleviate their health problems. Most participants received information on FGC-related health problems at seminars. |
