# Supplementary material for: Examining the key features of specialist health service provision for women with Female Genital Mutilation/Cutting (FGM/C) in the Global North: a scoping review
Source: Front Glob Womens Health. 2024 May 22;5:1329819. doi: 10.3389/fgwh.2024.1329819 (PMC11150566; doi:10.3389/fgwh.2024.1329819)
Supplement: Supplementary file 7 [file Datasheet3.pdf]

Supplementary File 3 - Search Strategy

Ovid MEDLINE(R) ALL <1946 to November 02, 2022>

#	Query	Results from 3 Nov 2022
1	female genital mutilation.ti,ab.	1,330
2	female genital cutting.ti,ab.	300
3	female circumcision.ti,ab.	432
4	FGM.ti,ab.	1,540
5	FGC.ti,ab.	480
6	(excision adj4 (wom?n or female* or girl*)).ti,ab.	784
7	(excision adj4 (genital* or clitor* or labia*)).ti,ab.	151
8	infibulation.ti,ab.	175
9	(ritual cutting adj (wom?n or female* or girl*)).ti,ab.	0
10	(ritual circumcision adj4 (wom?n or female* or girl*)).ti,ab.	2
11	pharaonic circumcision.ti,ab.	17
12	(sunna.mp. or sunnah circumcision.ti,ab.) adj4 (wom?n or female* or girl*).mp. [mp=title, book title, abstract, original title, name of substance word, subject heading word, floating sub-heading word, keyword heading word, organism supplementary concept word, protocol supplementary concept word, rare disease supplementary concept word, unique identifier, synonyms]	9
13	clitoridectom*.ti,ab.	98
14	1 or 2 or 3 or 4 or 5 or 6 or 7 or 8 or 9 or 10 or 11 or 12 or 13	3,981
15	(healthcare or health-care or health care or health).ti,ab.	2,490,440
16	(service* or model* or clinic*).ti,ab.	8,265,647
17	clinical practice.ti,ab.	232,722
18	(community or community-based).ti,ab.	591,378
19	holistic.ti,ab.	31,935

20	integrated.ti,ab.	300,360
21	(multi-disciplinary or multidisciplinary or interdisciplinary).ti,ab.	157,950
22	(provision or provider).ti,ab.	161,451
23	(medical or surgical or psychological).ti,ab.	2,555,273
24	(therapy or therapeutic).ti,ab.	2,988,173
25	treatment.ti,ab.	4,980,083
26	configuration*.ti,ab.	158,755
27	guideline*.ti,ab.	441,632
28	protocol*.ti,ab.	546,405
29	framework.ti,ab.	336,826
30	(theor* or theoretical).ti,ab.	768,015
31	speciali*.ti,ab.	270,161
32	referral*.ti,ab.	135,722
33	commission*.ti,ab.	47,676
34	access*.ti,ab.	639,378
35	intervention*.ti,ab.	1,239,012
36	reconstructi*.ti,ab.	286,852
37	care.ti,ab.	1,682,046
38	(deinfibulation or defibulation or reversal).ti,ab.	77,550
39	(wom?n centred or wom?n-centred or person centred or person-centred or patient centred or patient-centred).ti,ab.	11,224
40	hospital.ti,ab.	1,175,732
41	15 or 16 or 17 or 18 or 19 or 20 or 21 or 22 or 23 or 24 or 25 or 26 or 27 or 28 or 29 or 30 or 31 or 32 or 33 or 34 or 35 or 36 or 37 or 38 or 39 or 40	16,437,147
42	exp canada/ or exp mexico/ or exp united states/ or chile/ or Costa Rica/ or Colombia/ or israel/ or turkey/ or exp japan/ or exp Republic of Korea/ or austria/ or belgium/ or exp baltic states/ or estonia/ or latvia/ or lithuania/ or czech republic/ or hungary/ or poland/ or slovakia/ or slovenia/ or exp france/ or exp germany/ or united kingdom/ or exp england/ or northern ireland/ or exp scotland/ or wales/ or greece/ or exp ireland/ or exp italy/ or luxembourg/ or netherlands/ or portugal/ or exp "scandinavian and nordic countries"/ or exp denmark/ or finland/ or iceland/ or norway/ or sweden/ or spain/ or switzerland/ or exp australia/ or exp new zealand/ or (Australia or Austria or Belgium or Canada or Chile or	3,951,309

	Colombia or "Costa Rica" or "Czech Republic" or Denmark or Estonia or Finland or France or Germany or Greece or Hungary or Iceland or Israel or Italy or Japan or "Republic of Korea" or "South Korea" or Latvia or Lithuania or Luxembourg or Mexico or Netherlands or New Zealand or Norway or Poland or Portugal or "Slovak Republic" or Slovenia or Spain or Sweden or Switzerland or Turkey or "United Kingdom" or England or Ireland or Scotland or Wales or "United States").ti,ab.	
43	europe*.ti,ab.	338,391
44	(high-income or higher-income or high income or higher income).ti,ab.	20,834
45	42 or 43 or 44	4,167,264
46	14 and 41 and 45	628
47	limit 46 to yr="2012 - 2022"	390

https://ovidsp.ovid.com/ovidweb.cgi?T=JS&NEWS=N&PAGE=main&SHAREDSEARCHID=3BJ54qevGX4ClxgEX521bJai7BHFK8d1EAow20gFxnXoWSZc4PJJZuYEi0VFb2
