# Supplementary material for: Examining the key features of specialist health service provision for women with Female Genital Mutilation/Cutting (FGM/C) in the Global North: a scoping review
Source: Front Glob Womens Health. 2024 May 22;5:1329819. doi: 10.3389/fgwh.2024.1329819 (PMC11150566; doi:10.3389/fgwh.2024.1329819)
Supplement: Supplementary file 8 [file Datasheet4.pdf]

Supplementary File 5 - Data extraction sheet

**Study Characteristics**	Title
	1st author
	Year of publication
	Country in which the study conducted
	Type of publication (book chapter/journal article etc)
	Population/Number of participants
	Study design/Methodology
	Start date & End date
**Primary Key Features**	Whether described as specialist
	What interventions provided?
	What counselling provided?
	Is there a named service lead?
	Who provides care?
**Secondary Key Features /**<br>**(a) Context of care**	Eligibility
	Referral pathway
	Number of clients seen
	Cost
	Theoretical underpinnings
	When opened
	Whether advertised
**(b) Content of Care**	Model of care / care pathway
	Outcomes Measures (e.g. patient questionnaire/hcp questionnaire; validated or non-validated scale)
	Information / Education provided
**Summary of key features**	Offers R=Reconstuction, D=Deinfibulation, S=Sex therapy, T=Trauma therapy
	M=Maternity only; N=Non-pregnant only; B=Both pregnant & non-pregnant; NR=not reported
	MDT
	Is named as specialist care
	Has a designated service lead
	Only see women with FGM
	Does safeguarding assessment
	Education/Information provided
	Interpreter
	Community engagement
	Theoretical underpinning
